# Evaluation of GENESIS, SAIGE, REGENIE and fastGWA-GLMM for genome-wide association studies of binary traits in correlated data

**DOI:** 10.3389/fgene.2022.897210

**Published:** 2022-09-23

**Authors:** Anastasia Gurinovich, Mengze Li, Anastasia Leshchyk, Harold Bae, Zeyuan Song, Konstantin G. Arbeev, Marianne Nygaard, Mary F Feitosa, Thomas T Perls, Paola Sebastiani

**Affiliations:** ^1^ Institute for Clinical Research and Health Policy Studies, Tufts Medical Center, Boston, MA, United States; ^2^ Bioinformatics Program, Boston University, Boston, MA, United States; ^3^ Biostatistics Program, College of Public Health and Human Sciences, Oregon State University, Corvallis, OR, United States; ^4^ Department of Biostatistics, Boston University School of Public Health, Boston, MA, United States; ^5^ Biodemography of Aging Research Unit, Social Science Research Institute, Duke University, Durham, NC, United States; ^6^ Epidemiology, Biostatistics and Biodemography, Department of Public Health, University of Southern Denmark, Odense, Denmark; ^7^ Division of Statistical Genomics, Department of Genetics, Washington University School of Medicine, St Louis, MO, United States; ^8^ Department of Medicine, Geriatrics Section, Boston University School of Medicine, Boston, MA, United States

**Keywords:** GWAS, correlated data, binary phenotype, SPA, SAIGE, GENESIS, REGENIE, fastGWA-GLMM

## Abstract

Performing a genome-wide association study (GWAS) with a binary phenotype using family data is a challenging task. Using linear mixed effects models is typically unsuitable for binary traits, and numerical approximations of the likelihood function may not work well with rare genetic variants with small counts. Additionally, imbalance in the case-control ratios poses challenges as traditional statistical methods such as the Score test or Wald test perform poorly in this setting. In the last couple of years, several methods have been proposed to better approximate the likelihood function of a mixed effects logistic regression model that uses Saddle Point Approximation (SPA). SPA adjustment has recently been implemented in multiple software, including GENESIS, SAIGE, REGENIE and fastGWA-GLMM: four increasingly popular tools to perform GWAS of binary traits. We compare Score and SPA tests using real family data to evaluate computational efficiency and the agreement of the results. Additionally, we compare various ways to adjust for family relatedness, such as sparse and full genetic relationship matrices (GRM) and polygenic effect estimates. We use the New England Centenarian Study imputed genotype data and the Long Life Family Study whole-genome sequencing data and the binary phenotype of human extreme longevity to compare the agreement of the results and tools’ computational performance. The evaluation suggests that REGENIE might not be a good choice when analyzing correlated data of a small size. fastGWA-GLMM is the most computationally efficient compared to the other three tools, but it appears to be overly conservative when applied to family-based data. GENESIS, SAIGE and fastGWA-GLMM produced similar, although not identical, results, with SPA adjustment performing better than Score tests. Our evaluation also demonstrates the importance of adjusting by full GRM in highly correlated datasets when using GENESIS or SAIGE.

## Introduction

Mixed effects regression models are popular statistical models to analyze correlated data with multiple sources of variance, and their use in analyzing genome-wide association studies (GWAS) has increased, despite the computational challenges that they pose. To reduce the computational burden of mixed effects models, Chen et al. (Chen et al., 2016) proposed using a score test for testing the association between single nucleotide polymorphisms (SNP) and a binary trait in a GWAS. The score test uses the slope of the log-likelihood function evaluated under the null hypothesis for testing. The calculation of the score test requires fitting a single mixed effects model under the null hypothesis of no association. This model is the same for each variant across the genome and needs to be calculated only once. Testing the specific effect of a SNP requires weighting the residuals of this “null model” by the genotypes of each individual SNP. Fitting the “null model” requires to approximate the likelihood function using an iterative procedure, and Chen et al. proposed an approach based on the penalized quasi-likelihood method that is described in details in (H. Chen et al., 2016). This approach is implemented as the default choice for analysis of binary traits in the GENetic EStimation and Inference in Structured samples (GENESIS) R package (Gogarten et al., 2019). An advantage of the score test in comparison to the Wald and Likelihood ratio tests is that it does not require computing the maximum likelihood estimate for the genetic effect which could be computationally expensive for large datasets.

Recently, Zhou et al. proposed to apply the saddle point approximation (SPA) (Dey et al., 2017) to the score test in the GWAS setting (Zhou et al., 2018). In their paper, the authors noted that the normal approximation of the score test statistic becomes less accurate with increasing imbalance of cases and controls and with decreasing minor allele counts. While the normal approximation performs well near the mean of the distribution of the score test statistic, it performs very poorly at the tails and may lead to inaccurate *p*-values. The SPA method approximates the score test statistic by using the entire cumulant-generating function, rather than the first two moments (mean and variance) used with the normal approximation. The SPA adjustment has become very popular, and there are several programs that implement it, including the GENESIS R package, Scalable and Accurate Implementation of GEneralized mixed model (SAIGE) (Zhou et al., 2018), REGENIE (Mbatchou et al., 2021), and fastGWA-GLMM (Jiang et al., 2021). Another important challenge is how to adjust for the relatedness in family-based studies when using mixed-effects model. A popular approach that has replaced the use of the kinship matrix based on pedigree information is to use the estimated genetic relationship matrix (GRM), either sparse or full. The advantage of using the GRM is to leverage the genome-wide genotype data to estimate the kinship coefficients, rather than relying on reported familial relations (Wang & Thompson, 2019). GENESIS and SAIGE both have options to use either sparse or full GRMs. fastGWA-GLMM only has an option to adjust by a sparse GRM, and REGENIE uses the polygenic effect estimates to control for population and family structure.

A few studies compared statistical properties of these and other tools. For example, the work by Chen et al. (Chen et al., 2021) used simulated correlated data to assess Type I error rates and power of various implementations, while the works by Jiang et al. (Jiang et al., 2021) and Mbatchou et al. (Mbatchou et al., 2021) used biobank-scale datasets of unrelated individuals and simulated data with a small degree of relatedness. In this paper, we focus attention on assessing the agreement of the results as well as the efficiency of the implementations of a selection of tools using real correlated data. We compare implementations of the SPA to the score tests and the use of full versus sparse GRMs versus the polygenic effect estimates in GWASs of extreme longevity (EL) using imputed genotype data and whole-genome sequencing (WGS) data.

## Materials and methods

### Genetic data

New England Centenarian Study (NECS): This is a study of centenarians, with some family members and controls without parental longevity (Sebastiani & Perls, 2012). DNA collected in a subset of the participants was genotyped using Illumina SNP arrays and augmented with a selection of genome-wide genotype data from controls enrolled in various studies that were matched by genetic ethnicity (Sebastiani et al., 2012). Genome-wide genotype data of these subjects were imputed to the Haplotype Reference Consortium panel (version r1.1 2016) of 64,940 haplotypes with 39,635,008 sites using the Michigan Imputation Server as described in (Gurinovich et al., 2021). For the evaluation conducted in this work, we used the same case-control study of EL that we analyzed in Gurinovich et al. (Gurinovich et al., 2021) in which 1317 EL cases were individuals who survived beyond an age reached by less than 1% of individuals in their sex and birth-year cohort (males: 96 years for 1900, 97 years for 1910, 98 years for 1920; females: 100 years) based on the United States social security administration cohort tables (Bell & Miller, 2005), and 3,508 controls were genetically matched individuals without parental longevity.

Long Life Family Study (LLFS): This is a family-based study of longevity that enrolled 4,981 family members from 552 families selected for familial longevity between 2006 and 2009 (Wojczynski et al., 2022). DNA collected in a 3,681 subset of the participants were sequenced by the McDonnell Genome Institute (MGI) at Washington University in St. Louis via 150bp reads by Illumina Sequencers. Reads were aligned to build GRCh38 with BWA-MEM, marking duplicates with Picard, base quality score recalibration with Genome Analysis Toolkit (GATK), and lossless conversion to CRAM format with SAMtools. Variant calling was performed at the LLFS Data Coordinating Center, Division of Statistical Genomics at Washington University in St. Louis, using GATK4.1.0.0. GATK HaplotypeCaller was used to call variants from the CRAM files and create subject-level GVCF files. Next, these files were combined using GATK CombineGVCFs, and jointly-called using GATK GenotypeGVCFs. Finally, diallelic SNPs were extracted using GATK SelectVariants. QC steps included elimination of samples with insufficient haploid coverage and Mendelian inconsistencies, missing or monomorphic variants, excess heterozygosity (HETZ > .55 or p HETZ p< 1E-6) and call rate <0.9. For the evaluation conducted in this work, we identified 377 EL cases and 787 controls. EL cases were defined as in the NECS based on survival beyond an age reached by less than 1% of individuals in their sex and birth-year cohort.

### GWAS tools

Overall GWAS of EL set-up. The GWAS analyses were set up to account for relatedness in the data using mixed effects logistic regression. All the models were adjusted by sex and the first four genome-wide principal components (PCs). SNPs with minor allele count (MAC) < 3 in either cases or controls were removed from all the analyses. This threshold was adapted from the recent recommendation of including only SNPs with MAC > 5 (M. H. Chen et al., 2021).

GENESIS. To run the GWAS of EL with GENESIS, we used the nf-gwas-pipeline (https://github.com/montilab/nf-gwas-pipeline) (Song et al., 2021). GENESIS uses the genetic relationship matrix (GRM) to account for known and unknown relatedness in the data, and provides options to use the full and sparse GRMs. The pipeline consists of the following steps:

1. Files in the VCF format are converted to the GDS format which is defined by SeqArray (Zheng et al., 2017).
2. The snpgdsIBDKING() function from the SNPRelate R package (Manichaikul et al., 2010) is used to calculate the kinship matrix which is used in the next step.
3. The GENESIS functions pcair() (Conomos et al., 2015) and pcrelate() (Conomos et al., 2016) are used to calculate the genome-wide principal components and the GRM with the kinship threshold set to .2^−11/2^.This is the default value in the pcair() function, and it is used for declaring each pair of individuals as related or unrelated. A pre-defined subset of about 80,000 SNPs not in linkage disequilibrium (LD) was used for this step. This list of SNPs was derived by first excluding the SNPs in the two regions of high LD (MHC region on chromosome 6 and region of inversion polymorphism on chromosome 8) followed by iterative pruning of the genotype data to only keep independent SNPs. The SNP lists used in both, NECS and LLFS data can be found here: https://github.com/montilab/nf-gwas-pipeline/tree/master/data (NECS_plink_list.csv and LLFS_plink_list.csv files).
4. The null model is fit using only the fixed-effect covariates (sex and PCs) and the random effects are estimated. This step is conducted using the fitNullModel() function from the R package GENESIS.
5. Single-variant association tests are performed on the SNP data using either the Score or SPA-Score tests. This step uses the output from step 4 and assocTestSingle() function from the R package GENESIS. Note that the SPA adjustment is only applied to the SNPs whose *p*-values are less than 0.05 from the Score test.
6. Results are summarized via graphical displays (Manhattan and QQ plots) and SNPs are annotated using ANNOVAR (Wang et al., 2010).

The following versions of the software were used in these runs of the pipeline: R 4.1.1, GENESIS R package version 2.22.2, vcftools 0.1.16, bcftools 1.10.2, plink 2.00a1LM, annovar 2018apr, pandoc 2.5.

SAIGE. We used SAIGE version 1.0.5 (which at the time of the download was last updated on 1 April2,022) with R version 4.1.2 (2021-11-01). SAIGE assumes that the genome-wide principal components are provided as input. We used the same PCs that were calculated and used by the nf-gwas-pipeline. The SAIGE analysis consists of two steps:

1. Fit the null logistic mixed model to estimate the model parameters under the null hypothesis of no association including the GRM using step1_fitNULLGLMM.R script (https://saigegit.github.io//SAIGE-doc/docs/single_step1.html). For this step, we used the same lists of SNPs that were used in GENESIS functions pcair() and pcrelate().
2. Perform single SNP association tests using the step2_SPAtests.R script (https://saigegit.github.io//SAIGE-doc/docs/single_step2.html).

There is an additional step when using a sparse GRM in the SAIGE analysis. If a sparse GRM is specified, it needs to be calculated before step 1 above, which can be done using the createSparseGRM.R script (https://saigegit.github.io//SAIGE-doc/docs/createSparseGRM.html). In this case, unlike for the full GRM, the GRM does not need to be estimated in step 1 above. All scripts are provided by the developers of the package and can be installed using the instructions provided at the URL: https://saigegit.github.io//SAIGE-doc/docs/Installation.html. The scripts require specifying a series of input parameters. Supplementary Table S1 includes the complete list of input arguments to step1_fitNULLGLMM.R script. Supplementary Table S2 includes the complete list of input arguments to step2_SPAtests.R script. Supplementary Table S3 includes the complete list of input arguments to createSparseGRM.R script. The input arguments are essentially the same for the analysis of imputed dosages and WGS data, except for the vcfField argument. Some of the arguments in the Supplementary Tables 1 and 2 are required for using gene-based tests and are not used in the single-variant analysis, but are included in the supplement tables for the sake of reproducibility. One thing to note about SAIGE is that dropping samples with missing genotypes/dosages is not supported in the current version, so SAIGE imputes them as the best guessed.

REGENIE. REGENIE is a C++ program to perform a fast GWAS (Mbatchou et al., 2021). We used REGENIE version 3.1.1 in this analysis, and the same PCs were used as with GENESIS and SAIGE. The REGENIE analysis consists of two steps:

1. Fit the whole-genome regression model to the phenotype using a subset of SNPs to estimate a fraction of the phenotype variance. For this step, we used the same lists of SNPs that were used in GENESIS and SAIGE.
2. Perform single SNP association tests using a Firth logistic regression model conditional on the prediction from the model generated in step 1. For this step, we used Firth likelihood ratio test and SPA as fallback for p-values < 0.05

REGENIE can be downloaded from its GitHub page: https://github.com/rgcgithub/regenie and the documentation on how to use can be found here: https://rgcgithub.github.io/regenie/. One aspect to note about REGENIE is that it works with genotypes only, so the imputed dosages are converted to hard-call genotypes before association testing.

fastGWA-GLMM. fastGWA-GLMM is a tool that was recently implemented to run a fast and efficient GWAS (Jiang et al., 2021). It is a part of the GCTA software suit (Yang et al., 2011). We used GCTA version 1.94.0 beta. We used the same set of PCs as were used by all the other evaluated tools. fastGWA-GLMM consists of three steps:

1. Estimate full GRM (the full GRM cannot be used with the fastGWA-GLMM in the step 2 below) and generate sparse GRM from the full GRM at a cut-off value of 0.05. For this step, we used the same lists of SNPs that were used in all the other evaluated tools.
2. Estimate the variance component using fastGWA-B-REML algorithm (Jiang et al., 2021).
3. Perform single SNP association tests using the fastGWA-GLMM algorithm and the output from step 2.

The documentation for fastGWA-GLMM is available at https://yanglab.westlake.edu.cn/software/gcta/index.html#fastGWA-GLMM.

### Computational environment

All the analyses were performed using the Boston University Shared Computing Cluster. The platform is x86_64-pc-linux-gnu (64-bit) and the core operating system is CentOS Linux 7. The following resources were requested while submitting every script: a whole node was requested with 16 cores and at least 128 GB of RAM (-pe omp 16); a node that has at least 16GB of memory per core (-mem_per_core=16G) was used.

### Evaluation metrics

We report and compare results returned by SAIGE, GENESIS, fastGWA-GLMM and REGENIE using several metrics summarized in Table 1.

**TABLE 1:** Metrics to compare GWAS methods.

Metric	Description
Genomic inflation factor	The median of the observed chi-squared test statistics divided by the expected median of the corresponding chi-squared distribution
Agreement of the ranks of SNPs based on *p*-values	Test whether the rankings of the results are significantly different using two measures of rank correlation (Spearman’s Rho, Kendall’s Tau). The rank correlation coefficients were computed by setting different significance thresholds. (GENESIS and SAIGE only)
Agreement of the score statistics	Visual comparison, descriptive statistics (GENESIS, SAIGE and fastGWA-GLMM only)
Agreement of the effect estimates	Visual comparison, descriptive statistics
Computational resources	CPU time taken to calculate GRM matrices, run the null model and the per-chromosome association tests in the same computational environment

## Results

The results below are based on the 7,730,151 SNPs in the NECS imputed genotype data and 9,416,403 SNPs in the LLFS WGS data with MAC > 3 in either cases or controls in both datasets. We chose to use the MAC filter for both cases and controls instead of overall MAC filter as suggested in some studies (Zhou et al., 2018) (M. H. Chen et al., 2021), because we noticed that for some of the SNPs with MAC = 0 in either cases and controls, the SPA adjustment inflates the *p*-values and creates artifact significant results. This is only true for the SPA adjustment, and not for the Score test without the adjustment (Supplementary Figure S1).

**FIGURE 1:**
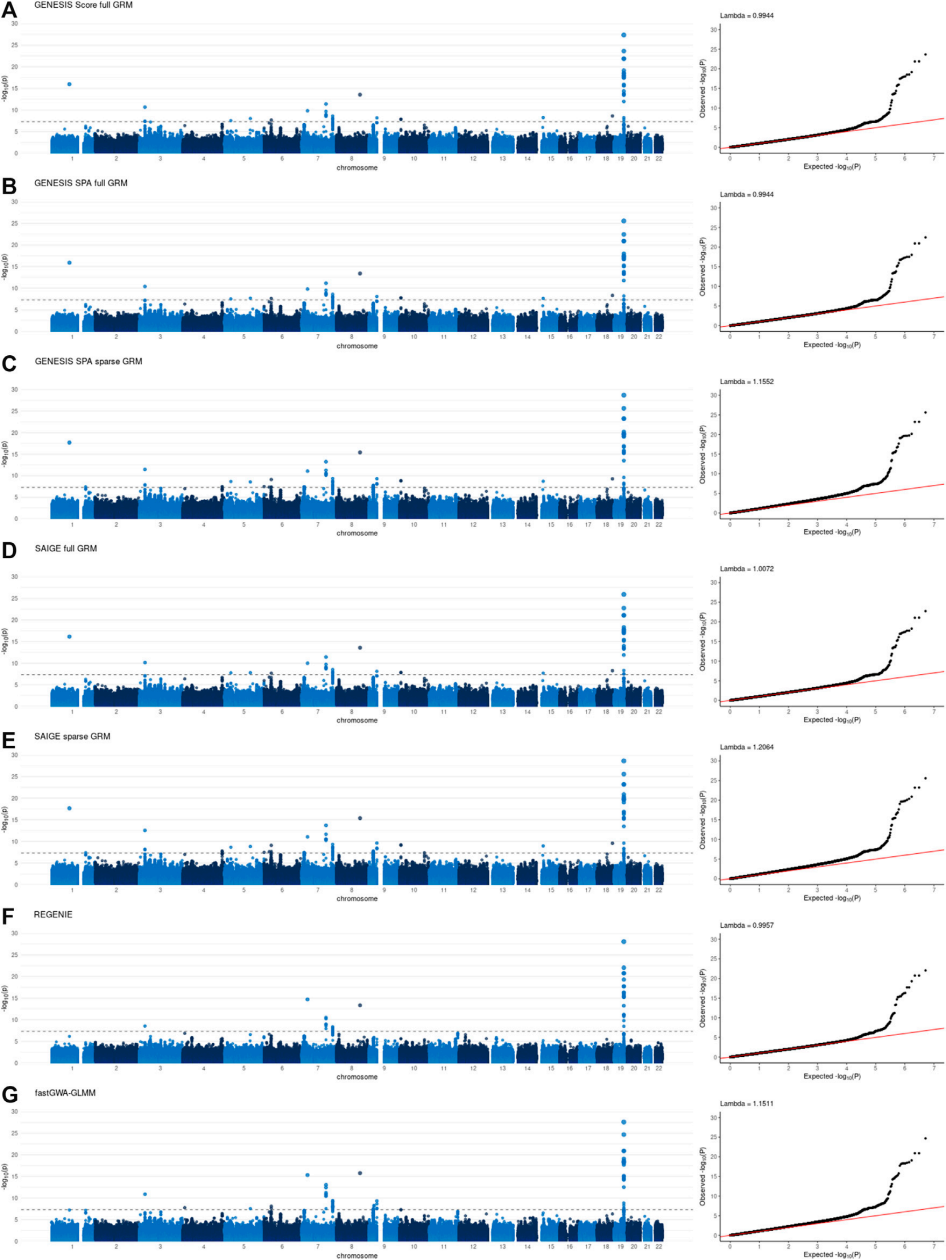
Manhattan and QQ plots of -log10(*p*-values) for the associations using imputed genotype data in the New England Centenarian Study (NECS) data. Panel **(A)**: associations based on the score test and adjusted for the full genetic relation matrix (GRM) using GENESIS. Panel **(B)**: associations based on the SPA and adjusted for the full GRM using GENESIS. Panel **(C)**: associations based on the SPA and adjusted for the sparse GRM using GENESIS. Panel **(D)**: associations based on the SPA and adjusted for the full GRM using SAIGE. Panel **(E)**: associations based on the SPA and adjusted for the sparse GRM using SAIGE. Panel **(F)**: associations based on the SPA and polygenic effect estimates to control for relatedness using REGENIE. Panel **(G)**: associations based on the SPA and adjusted for the sparse GRM using fastGWA-GLMM. Lambda is a genomic inflation factor.

**FIGURE 2:**
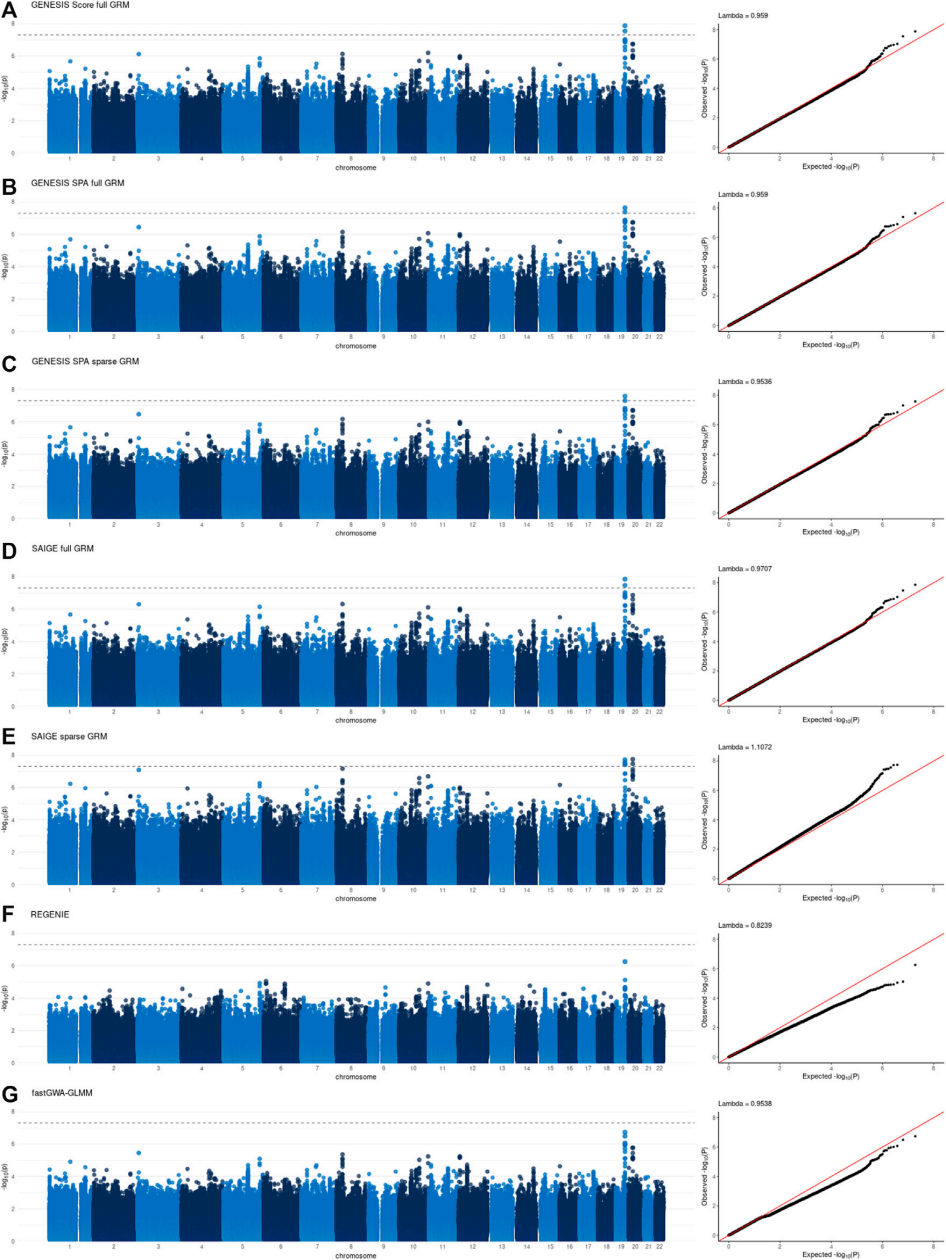
Manhattan and QQ plots of -log10(*p*-values) for the associations using WGS data in the Long Life Family Study (LLFS) data. Panel **(A)**: associations based on the score test and adjusted for the full genetic relation matrix (GRM) using GENESIS. Panel **(B)**: associations based on the SPA and adjusted for the full GRM using GENESIS. Panel **(C)**: associations based on the SPA and adjusted for the sparse GRM using GENESIS. Panel **(D)**: associations based on the SPA and adjusted for the full GRM using SAIGE. Panel **(E)**: associations based on the SPA and adjusted for the sparse GRM using SAIGE. Panel **(F)**: associations based on the SPA and polygenic effect estimates to control for relatedness using REGENIE. Panel **(G)**: associations based on the SPA and adjusted for the sparse GRM using fastGWA-GLMM. Lambda is a genomic inflation factor.

Individual GWAS results, specifically Manhattan and QQ plots, for all the tools are presented in Figures 1, 2 for NECS imputed genotype and LLFS WGS data respectively. Genomic inflation factors for each of the results can also be found in Figures 1, 2. SNPs that achieved genome-wide level of significance (*p*-value < 5 × 10^–8^) are presented in Supplementary Tables S4, S5. They replicate previous findings from the analyses that used the same or similar datasets (Sebastiani et al., 2017) (Gurinovich et al., 2021).

**FIGURE 3:**
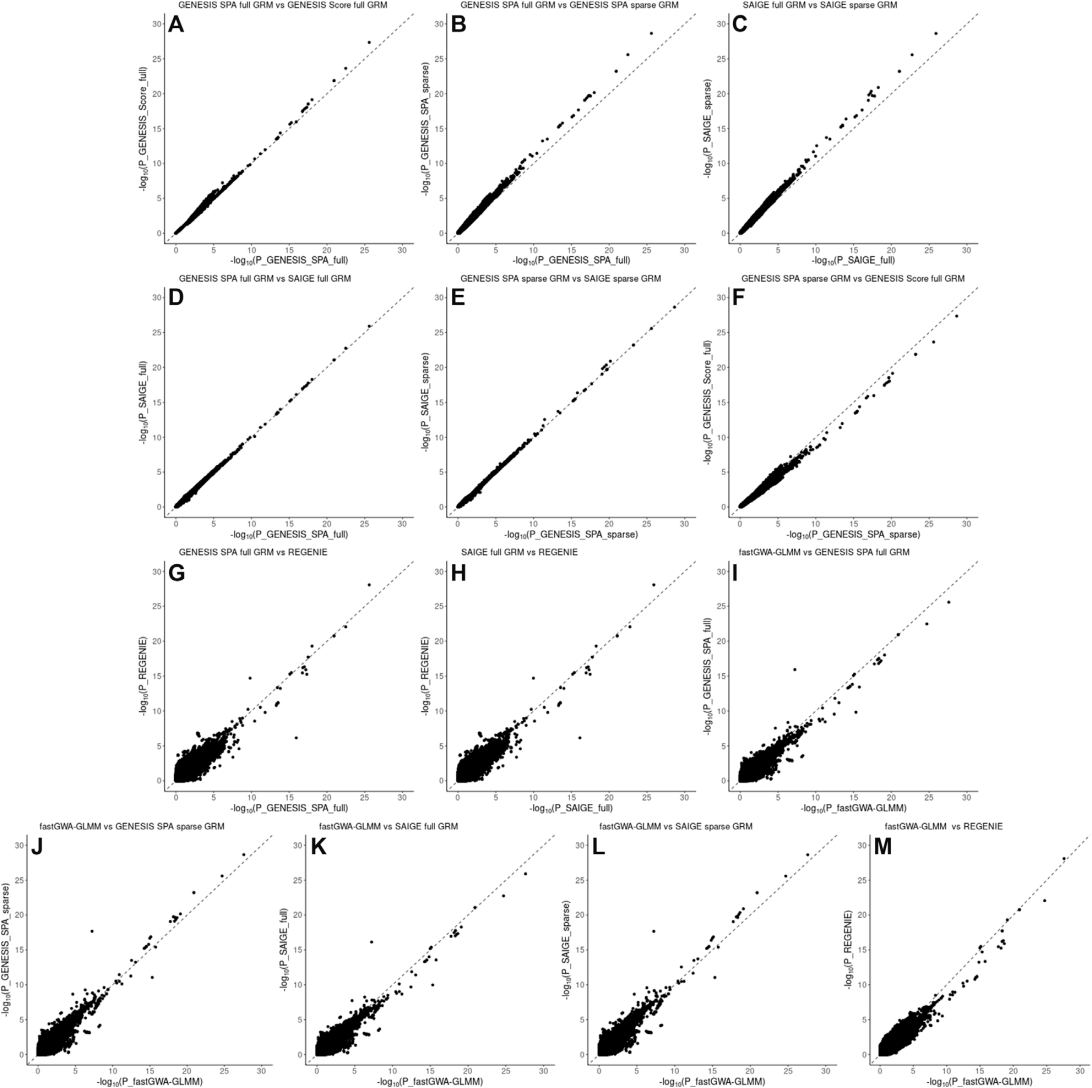
Pairwise comparison plots of –log10(*p* values) for the associations using imputed genotype data in the NECS data. Panel **(A)**: comparison of the associations based on the SPA and adjusted for the full genetic relation matrix (GRM) using GENESIS on the *X* axis versus associations based on the score test and adjusted for the full GRM using GENESIS on the *Y* axis. Panel **(B)**: comparison of the associations based on the SPA and adjusted for the full GRM using GENESIS on the *X* axis versus associations based on the SPA and adjusted for the sparse GRM using GENESIS on the *Y* axis. Panel **(C)**: comparison of the associations based on the SPA and adjusted for the full GRM using SAIGE on the *X* axis versus associations based on the SPA and adjusted for the sparse GRM using SAIGE on the *Y* axis. Panel **(D)**: comparison of the associations based on the SPA and adjusted for the full GRM using GENESIS on the *X* axis versus associations based on the SPA and adjusted for the full GRM using SAIGE on the *Y* axis. Panel **(E)**: comparison of the associations based on the SPA and adjusted for the sparse GRM using SAIGE on the *X* axis versus associations based on the SPA and adjusted for the sparse GRM using GENESIS on the *Y* axis. Panel **(F)**: comparison of the associations based on the SPA and adjusted for the sparse GRM using GENESIS on the *X* axis versus associations based on the score test and adjusted for the full GRM using GENESIS on the *Y* axis. Panel **(G)**: comparison of the associations based on the SPA and adjusted for the full GRM using GENESIS on the *X* axis versus associations based on the SPA and polygenic effect estimates to control for relatedness using REGENIE on the *Y* axis. Panel **(H)**: comparison of the associations based on the SPA and adjusted for the full GRM using SAIGE on the *X* axis versus associations based on the SPA and polygenic effect estimates to control for relatedness using REGENIE on the *Y* axis. Panel **(I)**: comparison of the associations based on the SPA and adjusted for the full GRM using fastGWA-GLMM on the *X* axis versus associations based on the SPA and adjusted for the full GRM using GENESIS on the *Y* axis. Panel **(J)**: comparison of the associations based on the SPA and adjusted for the full GRM using fastGWA-GLMM on the *X* axis versus associations based on the SPA and adjusted for the sparse GRM using GENESIS on the *Y* axis. Panel **(K)**: comparison of the associations based on the SPA and adjusted for the full GRM using fastGWA-GLMM on the *X* axis versus associations based on the SPA and adjusted for the full GRM using SAIGE on the *Y* axis. Panel (L): comparison of the associations based on the SPA and adjusted for the full GRM using fastGWA-GLMM on the *X* axis versus associations based on the SPA and adjusted for the sparse GRM using SAIGE on the *Y* axis. Panel (M): comparison of the associations based on the SPA and adjusted for the full GRM using fastGWA-GLMM on the *X* axis versus associations based on the SPA and polygenic effect estimates to control for relatedness using REGENIE on the *Y* axis.

**FIGURE 4:**
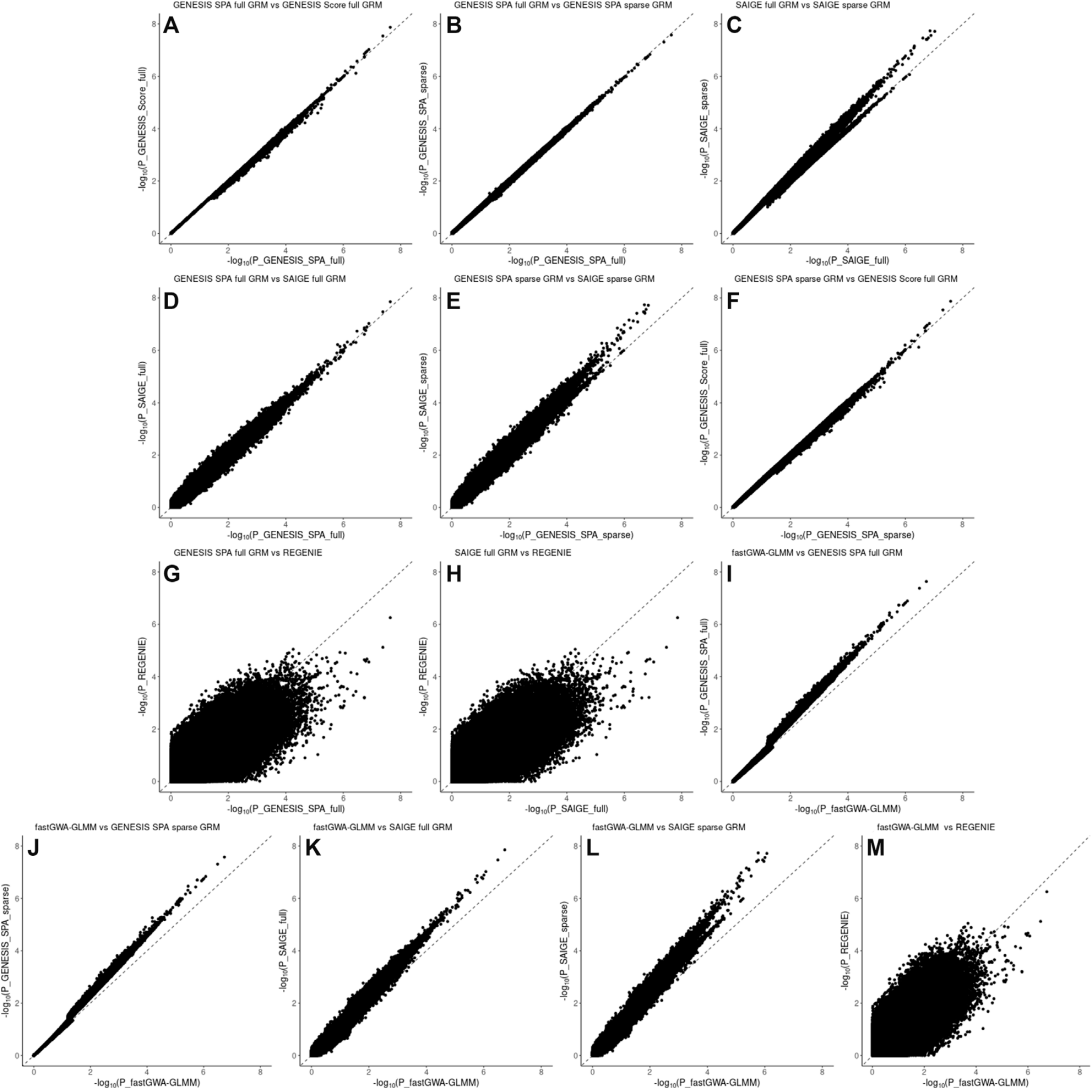
Pairwise comparison plots of –log10(pvalues) for the associations using WGS data in the LLFS data. Panel **(A)**: comparison of the associations based on the SPA and adjusted for the full genetic relation matrix (GRM) using GENESIS on the *X* axis versus associations based on the score test and adjusted for the full GRM using GENESIS on the *Y* axis. Panel **(B)**: comparison of the associations based on the SPA and adjusted for the full GRM using GENESIS on the *X* axis versus associations based on the SPA and adjusted for the sparse GRM using GENESIS on the *Y* axis. Panel **(C)**: comparison of the associations based on the SPA and adjusted for the full GRM using SAIGE on the *X* axis versus associations based on the SPA and adjusted for the sparse GRM using SAIGE on the *Y* axis. Panel **(D)**: comparison of the associations based on the SPA and adjusted for the full GRM using GENESIS on the *X* axis versus associations based on the SPA and adjusted for the full GRM using SAIGE on the *Y* axis. Panel **(E)**: comparison of the associations based on the SPA and adjusted for the sparse GRM using SAIGE on the *X* axis versus associations based on the SPA and adjusted for the sparse GRM using GENESIS on the *Y* axis. Panel **(F)**: comparison of the associations based on the SPA and adjusted for the sparse GRM using GENESIS on the *X* axis versus associations based on the score test and adjusted for the full GRM using GENESIS on the *Y* axis. Panel **(G)**: comparison of the associations based on the SPA and adjusted for the full GRM using GENESIS on the *X* axis versus associations based on the SPA and polygenic effect estimates to control for relatedness using REGENIE on the *Y* axis. Panel **(H)**: comparison of the associations based on the SPA and adjusted for the full GRM using SAIGE on the *X* axis versus associations based on the SPA and polygenic effect estimates to control for relatedness using REGENIE on the *Y* axis. Panel **(I)**: comparison of the associations based on the SPA and adjusted for the full GRM using fastGWA-GLMM on the *X* axis versus associations based on the SPA and adjusted for the full GRM using GENESIS on the *Y* axis. Panel **(J)**: comparison of the associations based on the SPA and adjusted for the full GRM using fastGWA-GLMM on the *X* axis versus associations based on the SPA and adjusted for the sparse GRM using GENESIS on the *Y* axis. Panel **(K)**: comparison of the associations based on the SPA and adjusted for the full GRM using fastGWA-GLMM on the *X* axis versus associations based on the SPA and adjusted for the full GRM using SAIGE on the *Y* axis. Panel (L): comparison of the associations based on the SPA and adjusted for the full GRM using fastGWA-GLMM on the *X* axis versus associations based on the SPA and adjusted for the sparse GRM using SAIGE on the *Y* axis. Panel (M): comparison of the associations based on the SPA and adjusted for the full GRM using fastGWA-GLMM on the *X* axis versus associations based on the SPA and polygenic effect estimates to control for relatedness using REGENIE on the *Y* axis.

Figure 3 and Figure 4, panel A confirm that using the SPA adjustment to the *p*-values from the score test makes the level of statistical significance more conservative, as shown by smaller −log_10_(p‐values) , although the magnitude of the correction varies with the degree of relatedness in the data. Using a sparse rather than a full GRM generally appears to inflate the statistical significance of the results based on the genomic inflation factors when applied to the data with a small degree of relatedness (NECS imputed genotype data) (Figure 1, panels C, E and G). When applied to family-based data (LLFS WGS data), GENESIS produced very similar results with either full or sparse GRMs (Figure 2, panels B and C), while SAIGE appears to produce more inflated *p*-values with sparse GRM as compared to the full GRM (Figure 2, panels E and D). REGENIE and fastGWA-GLMM produce rather conservative results with the LLFS WGS data (Figure 2, panels F and G). Overall, the results produced with GENESIS and SAIGE seem to be very similar when they both use the full GRM. The high similarity of the results is also confirmed by the descriptive statistics of the score function and effect estimates in Supplementary Table S6.

Pairwise comparison of the *p*-values for individual SNPs association with EL are presented in Figures 3, 4 for the NECS imputed genotype data and the LLFS WGS data respectively. These plots show that the SPA adjustment to the score test reduces the most extreme *p*-values (Figures 3, 4, panel A), and using the full GRM produces a more conservative correction of the *p*-values compared to using a sparse GRM in GENESIS and SAIGE in NECS imputed genotype data (Figure 3, panels B, C, F and K). The results of the SPA adjustment to the score tests with full GRM in GENESIS and SAIGE are very similar (Figures 3 and 4, panel D) but, when using a sparse GRM, SAIGE results appear to be less conservative than those produced by GENESIS (Figure 3 and Figure 4, panel E). Compared to GENESIS and SAIGE with full GRM, REGENIE appears to produce slightly more conservative *p*-values in NECS imputed genotype data (Figure 3, panels G and H) and substantially more conservative *p*-values in LLFS WGS data (Figure 4, panels G and H). Combined with the QQ-plot in Figure 2, panel F, the comparison suggests that REGENIE may over-correct *p*-values in family-based data. Finally, using a full GRM in SAIGE and GENESIS induces a more conservative correction of *p*-values compared to fastGWA-GLMM (Figure 3, panels I an K), but using a sparse GRM in SAIGE and GENESIS induces a less conservative correction of *p*-values compared to fastGWA-GLMM (Figure 3, panels J and L). Interestingly, with LLFS family-based data, fastGWA-GLMM produces substantially more conservative *p*-values than SAIGE and GENESIS with either sparse or full GRMs (Figure 4, panels I, J, K and L, panels). Combined with the QQ-plot in Figure 2, panel G, the comparison suggests that also fastGWA-GLMM may over-correct *p*-values in family-based data with possible loss of power.

To evaluate the similarity of the ranks of SNPs based on the extreme *p*-values in more details, we selected nested sets of SNPs with different levels of significance. In the GWAS with the NECS imputed genotype data, we used the following *p*-value thresholds: 5 × 10^−3^, 5 × 10^−4^, 5 × 10^−5^, 5 × 10^−6^, 5 × 10^−7^, and 5 × 10^−8^ (Table 2). In the GWAS with LLFS WGS data, we used the following *p*-value thresholds: 0.05, 5 × 10^−3^, 5 × 10^−4^, 5 × 10^−5^, and 5 × 10^−6^ (Table 3). The plots comparing the *p*-values of different methods and corresponding correlation coefficients can be found in Supplementary Figures S2, S3. Overall, the rank correlation coefficients were very high in both comparisons in both data sets. In the analysis of WGS data, inflation of test statistics was more evident in SAIGE with sparse GRM. Compared to the GENESIS SPA with sparse GRM, this inflation was also observed. No inflation was observed when comparing GENESIS SPA with full vs. sparse GRM. In the analysis of imputed genotype data, inflation of test statistics was observed for both methods using the sparse GRM. However, the methods using the same type of GRM (either full in both or sparse in both) produced very consistent results.

**TABLE 2:** Correlations of the ranks of the *p*-values for NECS imputed genotype data.

*p*-value threshold	Number of SNPs	GENESIS SPA full GRM vs. GENESIS score full GRM		GENESIS SPA full GRM vs. GENESIS SPA sparse GRM		SAIGE full GRM vs. SAIGE sparse GRM		GENESIS SPA full GRM vs. SAIGE full GRM		GENESIS SPA sparse GRM vs. SAIGE sparse GRM	
		Rho	Tau	Rho	Tau	Rho	Tau	Rho	Tau	Rho	Tau
5.00E-03	86,107	0.997	0.972	0.928	0.774	0.923	0.765	0.98	0.883	0.981	0.891
5.00E-04	13,054	0.983	0.935	0.92	0.763	0.912	0.747	0.975	0.871	0.98	0.892
5.00E-05	2,409	0.967	0.916	0.898	0.747	0.881	0.722	0.979	0.884	0.974	0.881
5.00E-06	609	0.976	0.936	0.946	0.822	0.949	0.828	0.984	0.909	0.983	0.917
5.00E-07	269	0.983	0.949	0.941	0.831	0.952	0.844	0.987	0.922	0.984	0.933
5.00E-08	100	0.984	0.952	0.917	0.817	0.937	0.831	0.991	0.952	0.985	0.941

Rho, Spearman’s Rho; Tau, Kendall’s Tau.

**TABLE 3:** Correlations of the ranks of the *p*-values for LLFS WGS data.

*p*-value threshold	Number of SNPs	GENESIS SPA full GRM vs. GENESIS score full GRM		GENESIS SPA full GRM vs. GENESIS SPA sparse GRM		SAIGE full GRM vs. SAIGE sparse GRM		GENESIS SPA full GRM vs. SAIGE full GRM		GENESIS SPA sparse GRM vs. SAIGE sparse GRM	
		Rho	Tau	Rho	Tau	Rho	Tau	Rho	Tau	Rho	Tau
5.00E-02	581,460	1	0.991	0.996	0.949	0.966	0.856	0.969	0.863	0.94	0.797
5.00E-03	68,524	0.996	0.958	0.995	0.94	0.912	0.771	0.954	0.83	0.877	0.707
5.00E-04	7,757	0.987	0.926	0.994	0.933	0.873	0.726	0.944	0.808	0.834	0.654
5.00E-05	840	0.986	0.917	0.994	0.938	0.791	0.637	0.949	0.816	0.767	0.589
5.00E-06	103	0.976	0.906	0.994	0.944	0.897	0.787	0.949	0.821	0.854	0.696

Rho, Spearman’s Rho; Tau, Kendall’s Tau.

There was no difference in the Score function values, and effect estimates between GENESIS Score and SPA tests as expected, as the SPA adjustments should be applied on the *p*-values only. There was a slight variation between GENESIS SPA and SAIGE’s Score function values, and a larger variation at the extremes of the Score function values calculated using the full and sparse GRMs in both GENESIS and SAIGE, but overall the results were very similar (Supplementary Figures S4, S5). There is a rather large and unexpected variation between GENESIS, SAIGE, fastGWA-GLMM and REGENIE in the effect estimates, but overall they were comparable (Supplementary Figures S6, S7).

Computational resources, such as CPU time, required by the whole nf-gwas-pipeline and SAIGE’s and REGENIE’s two steps are not directly comparable. Compared to SAIGE, REGENIE and fastGWA-GLMM, the nf-gwas-pipeline includes the additional step of calculating the genome-wide principal components. SAIGE, REGENIE and fastGWA-GLMM do not compute the principal components, but the user needs to provide them as input. We used principal components calculated using GENESIS as input to SAIGE, REGENIE and fastGWA-GLMM. Moreover, the four tools can take as input genetic data in different formats: GENESIS requires the GDS file format, SAIGE can use PLINK bed/bim/fam, VCF, BGEN, or SAV file formats, and REGENIE and fastGWA-GLMM can use the BGEN or PLINK (bed/bim/fam or pgen/pvar/psam) file formats. Conversion between various genetic data formats can be time consuming, and reading different data formats can take significantly different time which is not part of the actual GWAS analysis. We had our genetic data sets available in three file formats: GDS, PLINK bed/bim/fam, and VCF. It is possible that using other supported file formats (BGEN or SAVE for SAIGE and BGEN or PLINK pgen/pva/psam for REGENIE and fastGWA-GLMM) would take less or more time due to difference in times that it takes to read in different data files. We calculated the CPU time taken by GENESIS for the GRM calculation, the null model fitting and the association tests for chromosomes 1 and 21 using the GDS files as input. We also calculated the CPU time taken by SAIGE for the GRM calculation and the fitting of null model using PLINK bed/bim/fam files as input, and the association tests for chromosome 1 and 21 using the VCF files as input. We used VCF files as input to SAIGE because imputed genotype data includes dosages, and using PLINK bed/bim/fam file formats rounds the dosages to hard-call genotypes, and thus, loses on precision. We conducted an additional run of SAIGE using PLINK bed/bim/fam file format as input to all the steps using the WGS data to be able to compare it better with REGENIE and fastGWA-GLMM to remove the time difference of reading PLINK bed/bim/fam versus VCF file formats. We used PLINK bed/bim/fam file format as input to REGENIE and fastGWA-GLMM. Table 4 summarizes the times it took to run different steps and the overall time for all four tools. The CPU time of GENESIS SPA with full GRM was on average 1.06 fold the CPU time of GENESIS Score test with full GRM, and 1.19 fold the CPU time of GENESIS SPA with sparse GRM. The CPU time of SAIGE with sparse GRM was on average 1.09 fold the CPU time of SAIGE with full GRM. The CPU time of GENESIS SPA with full GRM was on average 2.11 fold SAIGE with full GRM, and the CPU time of GENESIS SPA with sparse GRM was on average 1.62 fold SAIGE with sparse GRM. The CPU time of SAIGE with full GRM using the PLINK bed/bim/fam file format was 0.18 fold the CPU time of SAIGE with full GRM using the VCF file format, and 0.29 fold the CPU time of REGENIE. FastGWA-GLMM was much faster than all the other tools: its CPU time ranged from 0.06 fold the CPU time of REGENIE to 0.16 fold the CPU time of SAIGE with full GRM using PLINK bed/bim/fam file format.

**TABLE 4:** Computation time used by GENESIS, SAIGE and REGENIE.

	Imputed dosages					WGS					
	GENESIS score	GENESIS SPA full / sparse	SAIGE full / sparse	REGENIE	fastGWA-GLMM	GENESIS score	GENESIS SPA full / sparse	SAIGE full / sparse	SAIGE full plink	REGENIE	fastGWA-GLMM
GRM calculations	1 h36 m32 s	1 h36 m32 s / 1 h35 m53 s	- / 0 h01 m38 s	-	0 h01 m50 s	2 h48 m36 s	2 h48 m36 s / 2 h48 m08 s	- / 0 h01 m04 s	-	-	0 h00 m12 s
Null model (and GRM calculations for SAIGE full GRM only)	0 h02 m57 s	0 h02 m56 s / 0 h00 m01 s	0 h20 m49 s / 0 h00 m10 s	0 h10 m06 s	0h 00m 04 s	0 h00 m13 s	0 h00 m06 s / 0 h00 m01 s	0 h04 m46 s / 0 h00 m04 s	0 h04 m46 s	0 h03 m18 s	0 h00 m02 s
Association tests chr1	1 h09 m40 s	1 h26 m58 s / 0 h41 m43 s	1 h00 m27 s / 1 h22 m59 s	0 h33 m02 s	0 h00 m59 s	0 h21 m05 s	0 h23 m46 s / 0 h20 m21 s	1 h12 m35 s / 1 h23 m42 s	0 h11 m19 s	0 h47 m56 s	0 h02 m00 s
						
Association tests chr21	0 h12 m59 s	0h16m09s / 0h06m22s	0 h13 m38 s / 0 h18 m23 s	0 h04 m34 s	0 h00 m10 s	0 h03 m00 s	0 h03 m22 s / 0 h03 m03 s	0 h16 m54 s / 0 h18 m34 s	0 h01 m06 s	0 h08 m00 s	0 h00 m21 s
						
Total time	3 h02 m08 s	3 h22 m35 s / 2 h23 m59 s	1 h34 m54 s / 1 h43 m10 s	0 h47 m42 s	0 h03 m03 s	3 h12 m54 s	3 h15 m50 s / 3 h11 m33 s	1 h34 m15 s / 1 h43 m24 s	0 h17 m11 s	0 h59 m14 s	0 h02 m35 s

GENESIS: Full and sparse GRM calculations are always done as a separate step. GDS file format is used as input for all the GWASs with GENESIS.

SAIGE: For the GWASs with full GRM, GRM calculation and null model fitting are combined in one step. For the GWASs with sparse GRM, the GRM is calculated in a separate step before fitting a null model. PLINK bed/bim/fam file format is used as input to GRM calculations and Null model steps for all the GWASs with SAIGE. SAIGE full / sparse GWASs use VCF file format as input for Association tests steps. SAIGE full plink GWAS uses PLINK bed/bim/fam file format as input for Association tests steps.

REGENIE: Null model step as referred to in here is fitting the whole-genome regression model to the phenotype in REGENIE GWASs. PLINK bed/bim/fam file format is used as input for both GWASs with REGENIE.

fastGWA-GLMM: Null model step as referred to in here is the estimation of variance component in fastGWA-GLMM. PLINK bed/bim/fam file format is used as input for both GWASs with fastGWA-GLMM.

## Discussion

We conducted a comparison of the implementation of the SPA of the score tests in GENESIS and SAIGE, and different ways to adjust for family relatedness using full or sparse GRMs as implemented in GENESIS, SAIGE and fast-GWA-GLMM, or polygenic effect estimates as implemented in REGENIE, using two real data sets with imputed dosages and WGS data. The comparisons suggest that GENESIS Score and SPA tests, SAIGE and fastGWA-GLMM produce comparable results, although there are some numerical differences. The SPA correction appears to reduce the statistical significance of SNP associations with the most extreme *p*-values. It has been shown from simulation studies that the Score test leads to inflated type 1 error rates for unbalanced case-control ratios at the genome-wide significance level, while type 1 error rates were well-controlled for the SPA method (Dey et al., 2017). Since thresholds for genome-wide significance are unambiguously adopted in GWAS, the use of SAIGE, GENESIS, and fastGWA-GLMM could produce inconsistent results for those associations with *p*-values close to genome-wide levels of significance. In addition, the difference between estimates that are derived from score function values have larger variability than the score function values themselves and should be interpreted with caution. Additionally, the comparison of using full versus sparse GRMs in both GENESIS SPA test and SAIGE also demonstrated the reduction of the inflation of significant results, but using a sparse GRM may not be effective with family-based data. FastGWA-GLMM appears to be more conservative than GENESIS and SAIGE with both sparse and full GRMs when used with the family-based data. This is an interesting observation, and requires more investigation, since, to the best of our knowledge, fastGWA-GLMM has not been evaluated with the family-based data before, and only with a simulated data with a small degree of relatedness (Jiang et al., 2021). The developers of REGENIE notice that the tool might produce too conservative results and might not be the best option to use on small data with highly correlated observations (Mbatchou et al., 2021). Our evaluation supports this observation and shows that the GWAS results can be too conservative especially in data sets with large families.

All four tools are extremely efficient; however, when comparing the same set of steps without accounting for the calculation of the genome-wide principal components and the generation and conversion of different file sets, fastGWA-GLMM is a clear winner and the fastest of all. The concern with fastGWA-GLMM is that, when applied to datasets with large number of related individuals, it appears to be very conservative.

In this work we focused on the efficiency and agreement of Score and SPA tests and various adjustments for relatedness as implemented by four popular tools: GENESIS, SAIGE, REGENIE and fastGWA-GLMM. A comprehensive assessment of the SPA to the score test was conducted in (Chen et al., 2021) using simulated data. The recommendation of the authors was that the SPA to the score test works well with a MAC > 5, although the developers of SAIGE recommend MAC > 20. We observed that, while it is important to use filters on very rare SNPs, in the analysis of binary phenotypes it is very important to use MAC filter in both cases and controls rather than an overall MAC filter, because having SNPs that are monomorphic in either cases or controls can introduce errors, and the inflation of *p*-values for these SNPs when using the SPA adjustment. Overall, our evaluation suggests that REGENIE and fastGWA-GLMM are overly conservative when used on small highly correlated datasets. SAIGE and GENESIS produce similar, but not equivalent, results, with SPA adjustment of the Score test and full GRM. Both tools perform well in small real correlated data, although SAIGE is more computationally efficient than GENESIS. fastGWA-GLMM is very fast and appears to work well in the datasets with a small number of related individuals. This observation is consistent with the conclusion in (Jiang et al., 2021). Advantage of the nf-gwas-pipeline, that uses GENESIS, is that, unlike SAIGE, REGENIE and fastGWA-GLMM, it also has an option to infer PCs and incorporate additional models, such as GMMAT, add SNP annotations and produce comprehensive reports (Song et al., 2021). However, it is not a specific advantage of GENESIS, but rather of other R packages that are part of the nf-gwas-pipeline.

## Data Availability

The LLFS data will be available through the ELITE portal to approved investigators. Requests to access the NECS dataset should be directed to the corresponding author.

## References

[B1] Bell F. Miller M. (2005). Life tables for the United States social security area 1900-2100. Washington, DC: Actuarial Study, 16.

[B2] Chen H. Wang C. Conomos M. P. Stilp A. M. Li Z. Sofer T. (2016). Control for population structure and relatedness for binary traits in genetic association studies via logistic mixed models. Am. J. Hum. Genet. 98 (4), 653–666. 10.1016/j.ajhg.2016.02.012 27018471 PMC4833218

[B3] Chen M. H. Pitsillides A. Yang Q. (2021). An evaluation of approaches for rare variant association analyses of binary traits in related samples. Sci. Rep. 11, 3145. 10.1038/s41598-021-82547-z 33542345 PMC7862354

[B4] Conomos M. P. Miller M. B. Thornton T. A. (2015). Robust inference of population structure for ancestry prediction and correction of stratification in the presence of relatedness. Genet. Epidemiol. 39 (4), 276–293. 10.1002/gepi.21896 25810074 PMC4836868

[B5] Conomos M. P. Reiner A. P. Weir B. S. Thornton T. A. (2016). Model-free estimation of recent genetic relatedness. Am. J. Hum. Genet. 98 (1), 127–148. 10.1016/j.ajhg.2015.11.022 26748516 PMC4716688

[B6] Dey R. Schmidt E. M. Abecasis G. R. Lee S. (2017). A fast and accurate algorithm to test for binary phenotypes and its application to PheWAS. Am. J. Hum. Genet. 101 (1), 37–49. 10.1016/j.ajhg.2017.05.014 28602423 PMC5501775

[B7] Gogarten S. M. Sofer T. Chen H. Yu C. Brody J. A. Thornton T. A. (2019). Genetic association testing using the GENESIS R/Bioconductor package. Bioinforma. Oxf. Engl. 35 (24), 5346–5348. 10.1093/bioinformatics/btz567 PMC7904076 31329242

[B8] Gurinovich A. Song Z. Zhang W. Federico A. Monti S. Andersen S. L. (2021). Effect of longevity genetic variants on the molecular aging rate. GeroScience 43 (3), 1237–1251. 10.1007/s11357-021-00376-4 33948810 PMC8190315

[B9] Jiang L. Zheng Z. Fang H. Yang J. (2021). A generalized linear mixed model association tool for biobank-scale data. Nat. Genet. 53 (11), 1616–1621. 10.1038/s41588-021-00954-4 34737426

[B10] Manichaikul A. Mychaleckyj J. C. Rich S. S. Daly K. Sale M. Chen W. M. (2010). Robust relationship inference in genome-wide association studies. Bioinformatics 26 (22), 2867–2873. 10.1093/bioinformatics/btq559 20926424 PMC3025716

[B11] Mbatchou J. Barnard L. Backman J. Marcketta A. Kosmicki J. A. Ziyatdinov A. (2021). Computationally efficient whole-genome regression for quantitative and binary traits. Nat. Genet. 53 (7), 1097–1103. 10.1038/s41588-021-00870-7 34017140

[B12] Sebastiani P. Solovieff N. DeWan A. T. Walsh K. M. Puca A. Hartley S. W. (2012). Genetic signatures of exceptional longevity in humans. PLOS ONE 7 (1), e29848–22. 10.1371/journal.pone.0029848 22279548 PMC3261167

[B13] Sebastiani P. Perls T. T. (2012). The genetics of extreme longevity: Lessons from the new England centenarian study. Front. Genet. 3 (277). 10.3389/fgene.2012.00277 PMC3510428 23226160

[B14] Sebastiani P. Gurinovich A. Bae H. Andersen S. Malovini A. Atzmon G. (2017). Four genome-wide association studies identify new extreme longevity variants. J. Gerontol. A Biol. Sci. Med. Sci. 72 (11), 1453–1464. 10.1093/gerona/glx027 28329165 PMC5861867

[B15] Song Z. Gurinovich A. Federico A. Monti S. Sebastiani P. (2021). Nf-gwas-pipeline: A nextflow genome-wide association study pipeline. J. Open Source Softw. 6 (59), 2957. 10.21105/joss.02957 35647481 PMC9137404

[B16] Wang B. Thompson E. (2019). Realized genome sharing in heritability estimation using random effects models. G3 9 (5), 1385–1391. 10.1534/g3.119.0005 30902892 PMC6505141

[B17] Wang K. Li M. Hakonarson H. (2010). Annovar: Functional annotation of genetic variants from high-throughput sequencing data. Nucleic Acids Res. 38 (16), e164–7. 10.1093/nar/gkq603 20601685 PMC2938201

[B18] Wojczynski M. K. Jiuan Lin S. Sebastiani P. Perls T. T. Lee J. Kulminski A. (2022). NIA Long Life family study: Objectives, design, and heritability of cross-sectional and longitudinal phenotypes. J. Gerontol. A Biol. Sci. Med. Sci. 77 (4), 717–727. 10.1093/gerona/glab333 34739053 PMC8974329

[B19] Yang J. Lee S. H. Goddard M. E. Visscher P. M. (2011). Gcta: A tool for genome-wide complex trait analysis. Am. J. Hum. Genet. 88 (1), 76–82. 10.1016/j.ajhg.2010.11.011 21167468 PMC3014363

[B20] Zheng X. Gogarten S. M. Lawrence M. Stilp A. Conomos M. P. Weir B. S. (2017). SeqArray-a storage-efficient high-performance data format for WGS variant calls. Bioinformatics 33 (15), 2251–2257. 10.1093/bioinformatics/btx145 28334390 PMC5860110

[B21] Zhou W. Nielsen J. B. Fritsche L. G. Dey R. Gabrielsen M. E. Wolford B. N. (2018). Efficiently controlling for case-control imbalance and sample relatedness in large-scale genetic association studies. Nat. Genet. 50 (9), 1335–1341. 10.1038/s41588-018-0184-y 30104761 PMC6119127

