# Supplementary material for: Evaluation of GENESIS, SAIGE, REGENIE and fastGWA-GLMM for genome-wide association studies of binary traits in correlated data
Source: Front Genet. 2022 Sep 23;13:897210. doi: 10.3389/fgene.2022.897210 (PMC9544087; doi:10.3389/fgene.2022.897210)

Supplementary Table 1. List of input arguments to the step1_fitNULLGLMM.R SAIGE script

The values defined by the user (not default) are typed in bold.

SAIGE input argument names	SAIGE input argument values
**plinkFile**	Input plink file
**phenoFile**	Input phenotype file
**phenoCol**	Column name in the phenotype file with the binary phenotype to be tested
**traitType**	"binary"
invNormalize	FALSE
**covarColList**	Column names in the phenotype file which to be included as fixed covariates in the model ("Sex,PC1,PC2,PC3,PC4")
**qCovarColList**	“Sex”
**sampleIDColinphenoFile**	Column name in the phenotype file which contains sample ids
tol	0.02
maxiter	20
tolPCG	1e-05
maxiterPCG	500
**nThreads**	24 for full GRM; 1 for sparse GRM
SPAcutoff	2
numRandomMarkerforVarianceRatio	30
skipModelFitting	FALSE
**skipVarianceRatioEstimation**	FALSE
memoryChunk	2
tauInit	"0,0"
LOCO	TRUE for full GRM; FALSE for sparse GRM
traceCVcutoff	0.0025
ratioCVcutoff	0.001
**outputPrefix**	Prefix of the output files to be generated in this step

outputPrefix_varRatio	""
**IsOverwriteVarianceRatioFile**	TRUE
**sparseGRMFile**	"" for full GRM; link to a sparse GRM file calculated using createSparseGRM.R script for sparse GRM
**sparseGRMSampleIDFile**	"" for full GRM; link to a sparse GRM sample id file calculated using createSparseGRM.R script for sparse GRM
isCateVarianceRatio	FALSE
relatednessCutoff	0
cateVarRatioMinMACVecExclude	"10,20.5"
cateVarRatioMaxMACVecInclude	"20.5"
isCovariateTransform	TRUE
isDiagofKinSetAsOne	FALSE
**useSparseGRMtoFitNULL**	FALSE for full GRM; TRUE for sparse GRM
useSparseGRMforVarRatio	FALSE
minMAFforGRM	0.01
maxMissingRateforGRM	0.15
minCovariateCount	-1
includeNonautoMarkersforVarRatio	FALSE
FemaleOnly	FALSE
MaleOnly	FALSE
sexCol	""
FemaleCode	"1"
MaleCode	"0"
isCovariateOffset	TRUE
SampleIDIncludeFile	""
help	FALSE

Supplementary Table 2. List of input arguments to the step2_SPAtests.R SAIGE script

The values defined by the user (not default) are typed in bold.

SAIGE input argument names	SAIGE input argument values

**vcfFile**	Input VCF file (.vcf.gz)
**vcfFileIndex**	Input VCF index file (.vcf.gz.csi)
**vcfField**	DS (for imputed genotype data)/GT (for WGS genotypes)
bgenFile	""
bgenFileIndex	""
savFile	""
savFileIndex	""
sampleFile	""
bedFile	""
bimFile	""
famFile	""
AlleleOrder	"alt-first"
idstoIncludeFile	""
rangestoIncludeFile	""
**chrom**	Chromosome to be analyzed
is_imputed_data	FALSE
minMAF	0
minMAC	0.5
minGroupMAC_in_BurdenTest	5
minInfo	0
maxMissing	0.15
impute_method	"best_guess"
**GMMATmodelFile**	Model file from step 1 (step1_fitNULLGLMM.R)
**varianceRatioFile**	Variance ratio file from step 1 (step1_fitNULLGLMM.R)
**SAIGEOutputFile**	A file with association test results to be generated in this step
**LOCO**	TRUE for full GRM; FALSE for sparse GRM
markers_per_chunk	10000
groups_per_chunk	100

**is_output_moreDetails**	TRUE
is_overwrite_output	TRUE
maxMAF_in_groupTest	"0.0001,0.001,0.01"
maxMAC_in_groupTest	"0"
annotation_in_groupTest	"lof,missense;lof,missense;lof;synonymous"
groupFile	""
**sparseGRMFile**	"" for full GRM; link to a sparse GRM file calculated using createSparseGRM.R script for sparse GRM
**sparseGRMSampleIDFile**	"" for full GRM; link to a sparse GRM sample id file calculated using createSparseGRM.R script for sparse GRM
relatednessCutoff	0
MACCutoff_to_CollapseUltraRare	10
cateVarRatioMinMACVecExclude	"10,20.5"
cateVarRatioMaxMACVecInclude	"20.5"
weights.beta	"1,25"
r.corr	"0"
markers_per_chunk_in_groupTest	100
condition	""
SPAcutoff	2
dosage_zerod_cutoff	0.2
dosage_zerod_MAC_cutoff	10
is_single_in_groupTest	FALSE
is_no_weight_in_groupTest	FALSE
is_output_markerList_in_groupTest	FALSE
**is_Firth_beta**	TRUE
**pCutoffforFirth**	0.01
help	FALSE

Supplementary Table 3. List of input arguments to the createSparseGRM.R script

The values defined by the user (not default) are typed in bold.

**SAIGE input argument names**	**SAIGE input argument values for imputed dosages and WGS data analyses**
**plinkFile**	Input plink file
**nThreads**	4
memoryChunk	2
**outputPrefix**	Prefix of the output files to be generated in this step
**numRandomMarkerforSparseKin**	2000
**relatednessCutoff**	0.125
isDiagofKinSetAsOne	FALSE
minMAFforGRM	0.01
maxMissingRateforGRM	0.15
help	FALSE

Supplementary Table 4. Genome-wide level significant results using imputed genotype data

rsid	Gene	Consequence	Allele frequency	p.value genesis_score	p.value genesis_spa_full	p.value genesis_spa_sparse	p.value saige_full	p.value saige_sparse	p.value regenie	p.value fastGWA-GLMM
rs7521051	LPHN2	intron variant	0.389732	1.08E-16	1.18E-16	2.12E-18	7.52E-17	2.17E-18	7.18E-07	0.00000006
rs78043944	-	intergenic variant	0.0105434	2.17E-11	3.74E-11	3.55E-12	7.68E-11	2.85E-13	2.85E-09	1.30E-11
rs649357	-	intergenic variant	0.62335	2.89E-08	2.86E-08	2.22E-09	1.87E-08	2.25E-09	4.10E-04	0.00002
rs1961191	RNU6-373P	upstream gene variant	0.00594611	9.42E-09	1.93E-08	2.71E-09	1.65E-08	1.49E-09	2.70E-07	0.00000003
rs1042151	HLA-DPB1	missense variant	0.0880647	2.11E-08	2.20E-08	7.82E-10	2.10E-08	8.16E-10	5.75E-07	0.000000008
rs1554639	-	intergenic variant	0.634122	1.52E-10	1.51E-10	8.84E-12	1.07E-10	9.09E-12	2.13E-15	4.83E-16
rs6977506	DOCK4	intron variant	0.0972286	4.05E-12	6.63E-12	5.88E-14	3.93E-12	1.96E-14	3.04E-11	8.61E-14
rs1987475	TRBV6-6	upstream gene variant	0.680713	2.36E-09	2.34E-09	4.74E-10	3.19E-09	4.83E-10	4.55E-09	4.13E-10
rs2449839	-	intergenic variant	0.712167	2.83E-14	3.74E-14	4.06E-16	2.77E-14	4.13E-16	4.81E-14	1.71E-16
rs10973748	-	intergenic variant	0.0387546	6.57E-09	8.01E-09	5.08E-10	8.02E-09	2.40E-10	2.99E-06	4.53E-10
rs12266675	-	intergenic variant	0.115198	1.43E-08	1.65E-08	1.50E-09	1.54E-08	6.96E-10	2.65E-06	0.00000005
rs77184423	APBA2	intron variant	0.0075428	5.69E-09	2.18E-08	1.98E-09	2.09E-08	1.10E-09	6.54E-06	0.0000003
rs77546126	-	intergenic variant	0.0046886	2.35E-09	4.45E-09	5.40E-10	5.84E-09	2.70E-10	2.53E-05	0.0000005
rs429358	APOE / TOMM40	missense variant	0.11502	4.40E-28	2.57E-26	2.24E-29	1.24E-26	2.30E-29	1.56E-28	2.05E-31

Supplementary Table 5. Genome-wide level significant results using WGS data

rsid	Gene	Consequence	Allele frequency	p.value genesis_score	p.value genesis_spa_full	p.value genesis_spa_sparse	p.value saige_full	p.value saige_sparse	p.value regenie	p.value fastGWA-GLMM
rs429358	APOE	missense variant	0.113832	1.34E-08	2.31E-08	2.65E-08	1.40E-08	2.61E-09	5.69E-07	0.0000001

Supplementary Table 6. Descriptive statistics for the score function values and effect estimates for GENESIS Score/SPA, SAIGE, REGENIE, and fastGWA-GLMM

Note: REGENIE does not have Score function values

Statistic		Imputed dosages					WGS				
		**GENESIS Score**	**GENESIS SPA full / sparse**	**SAIGE full / sparse**	**REGENIE**	**fastGWA-GLMM**	**GENESIS Score**	**GENESIS SPA full / sparse**	**SAIGE full / sparse**	**REGENIE**	**fastGWA-GLMM**
Score	min	-155	-155 / -174	-156 / -174	*<NA>*	-172.	-49.1	-49.1 / -48.9	-49.6 / -49.4	*<NA>*	-49.0
	median	0.267	0.267 / 0.303	0.268 / 0.304	*<NA>*	0.36	0.1999	0.199 / 0.198	0.211 / 0.21	*<NA>*	0.198
	mean	0.0373	0.0373 / 0.0333	0.0356 / 0.0333	*<NA>*	0.0296	0.0895	0.0895 / 0.0889	0.104 / 0.104	*<NA>*	0.0890
	max	138	138 / 158	139 / 158	*<NA>*	118.	44	44 / 43.8	45 / 44.8	*<NA>*	43.8
Effect estimate	min	-3.57	-3.57 / -3.81	-2.69 / -3.02	-2.36	-3.27	-2.22	-2.22 / -2.2	-2.09 / -2.09	-4.31	-2.14
	median	0.00504	0.00504 / 0.00512	0.00505 / 0.00536	0.00521	0.00565	0.0115	0.0115 / 0.0115	0.0121 / 0.0138	0.00864	0.0115
	mean	0.04	0.04 / 0.043	0.0397 / 0.0434	0.0366	0.0431	0.0426	0.00426 / 0.0425	0.0423 / 0.0485	0.0344	0.0414

	max	4.57	4.57 / 5.38	3.93 / 4.23	3.09	4.33	2.56	2.56 / 2.58	2.55 / 2.55	3.50	2.45

Supplementary Figure 1.

(A) GENESIS Score, MAC > 5 vs MAC > 3 in cases and controls

MAC > 5

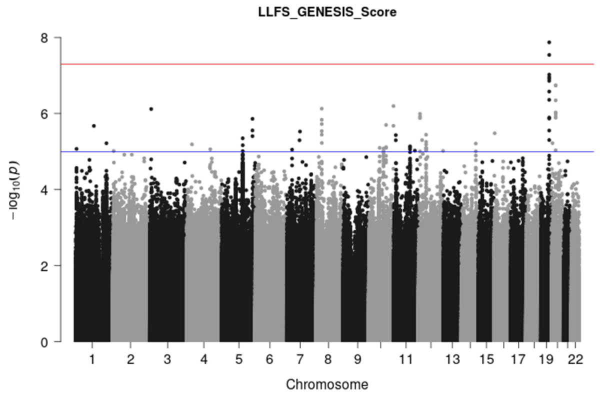

MAC > 3 in cases and controls

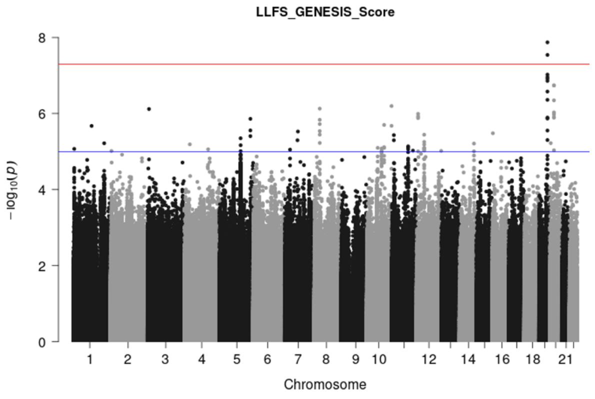

(B) GENESIS SPA full GRM, MAC > 5 vs MAC > 3 in cases and controls

MAC > 5

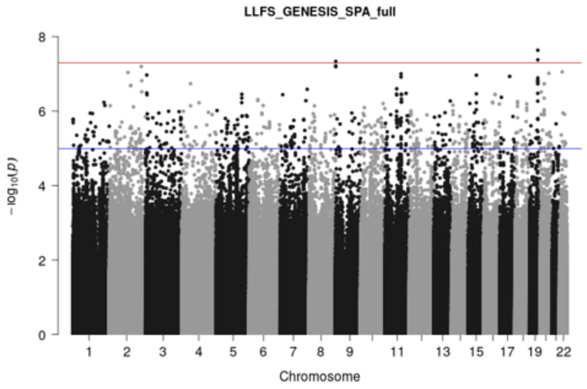

MAC > 3 in cases and controls

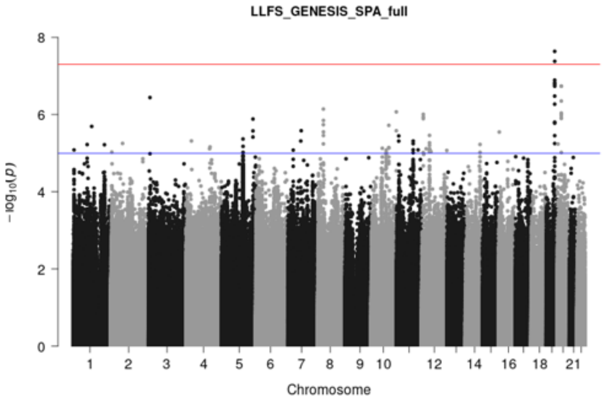

(C) SAIGE full GRM, MAC > 5 vs MAC > 3 in cases and controls

MAC > 5

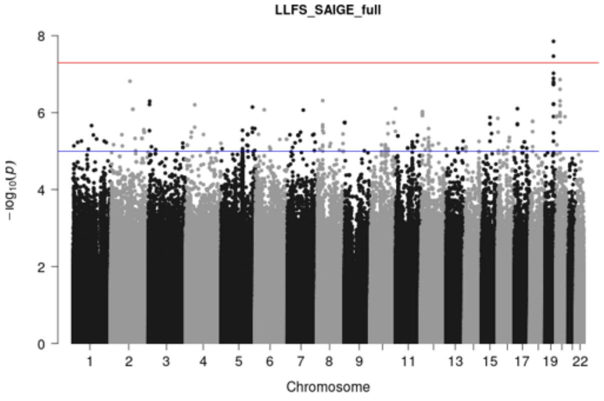

MAC > 3 in cases and controls

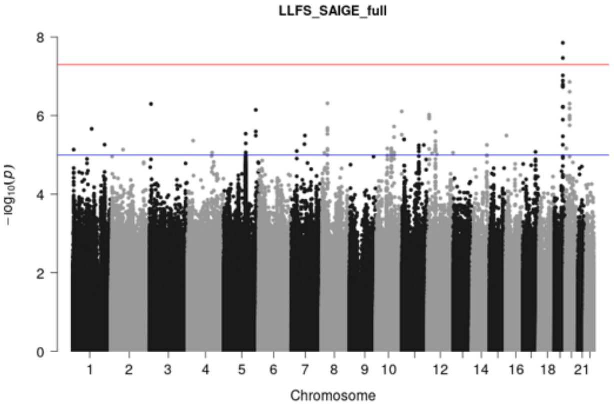

Supplementary Figure 2. Correlations of the ranks of the p-values for imputed genotype data

(A)

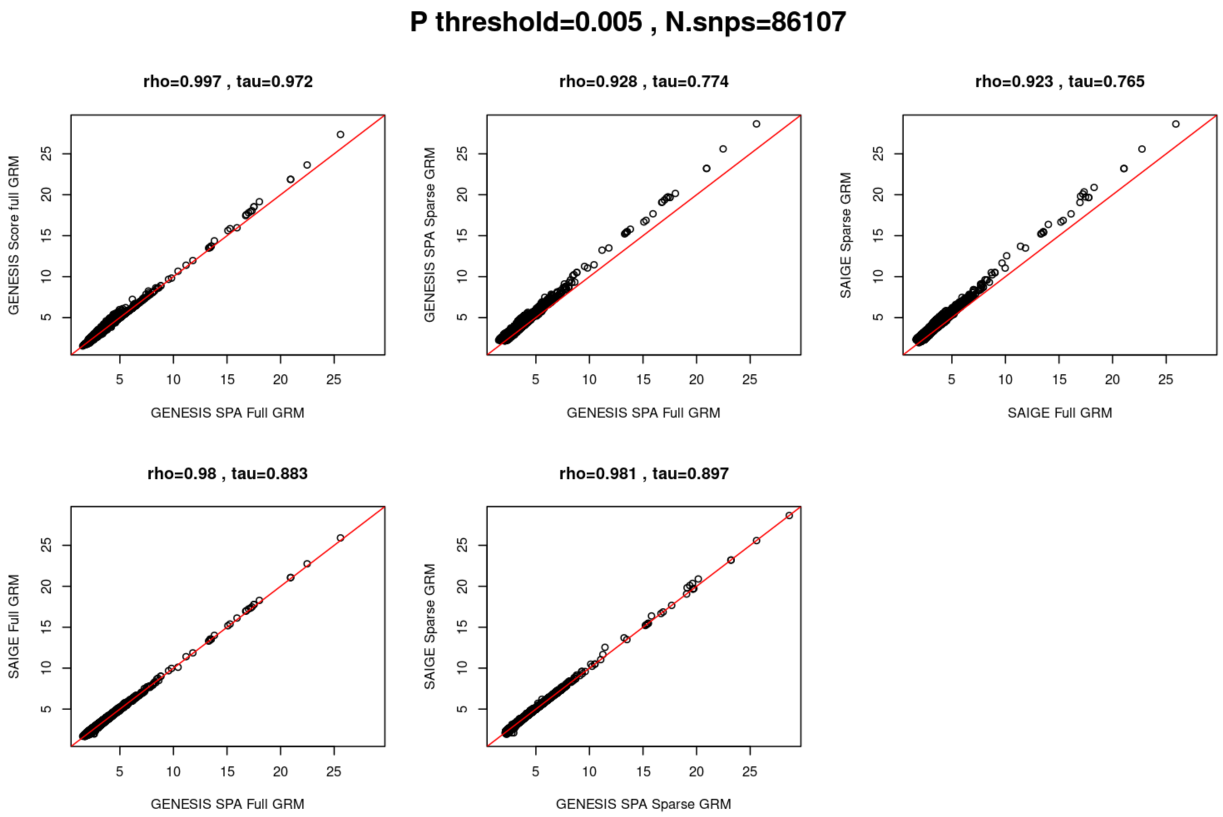
P threshold=0.005 , N.snps=86107
rho=0.997 , tau=0.972
GENESIS Score full GRM
GENESIS SPA Full GRM
rho=0.928 , tau=0.774
GENESIS SPA Sparse GRM
GENESIS SPA Full GRM
rho=0.923 , tau=0.765
SAIGE Sparse GRM
SAIGE Full GRM
rho=0.98 , tau=0.883
SAIGE Full GRM
GENESIS SPA Full GRM
rho=0.981 , tau=0.897
SAIGE Sparse GRM
GENESIS SPA Sparse GRM

(B)

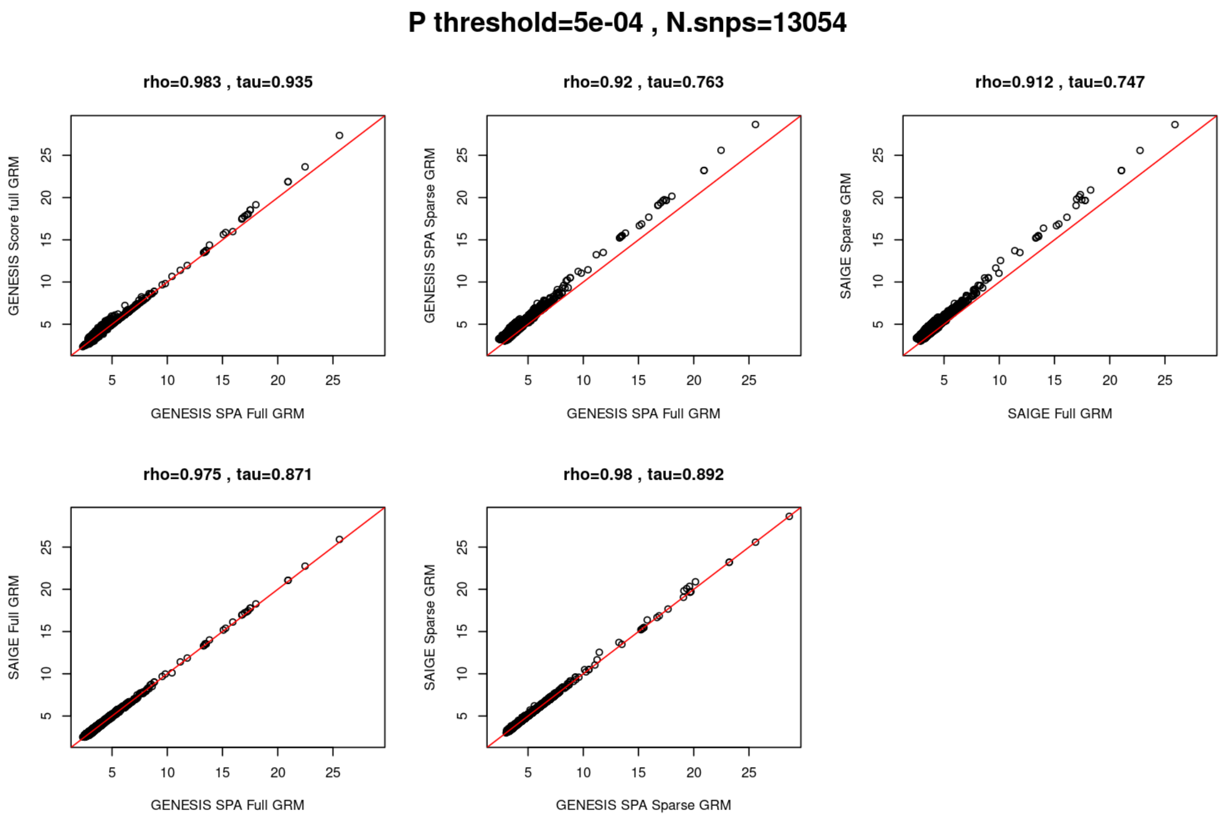
P threshold=5e-04 , N.snps=13054
rho=0.983 , tau=0.935
GENESIS Score full GRM
GENESIS SPA Full GRM
rho=0.92 , tau=0.763
GENESIS SPA Sparse GRM
GENESIS SPA Full GRM
rho=0.912 , tau=0.747
SAIGE Sparse GRM
SAIGE Full GRM
rho=0.975 , tau=0.871
SAIGE Full GRM
GENESIS SPA Full GRM
rho=0.98 , tau=0.892
SAIGE Sparse GRM
GENESIS SPA Sparse GRM

(C)

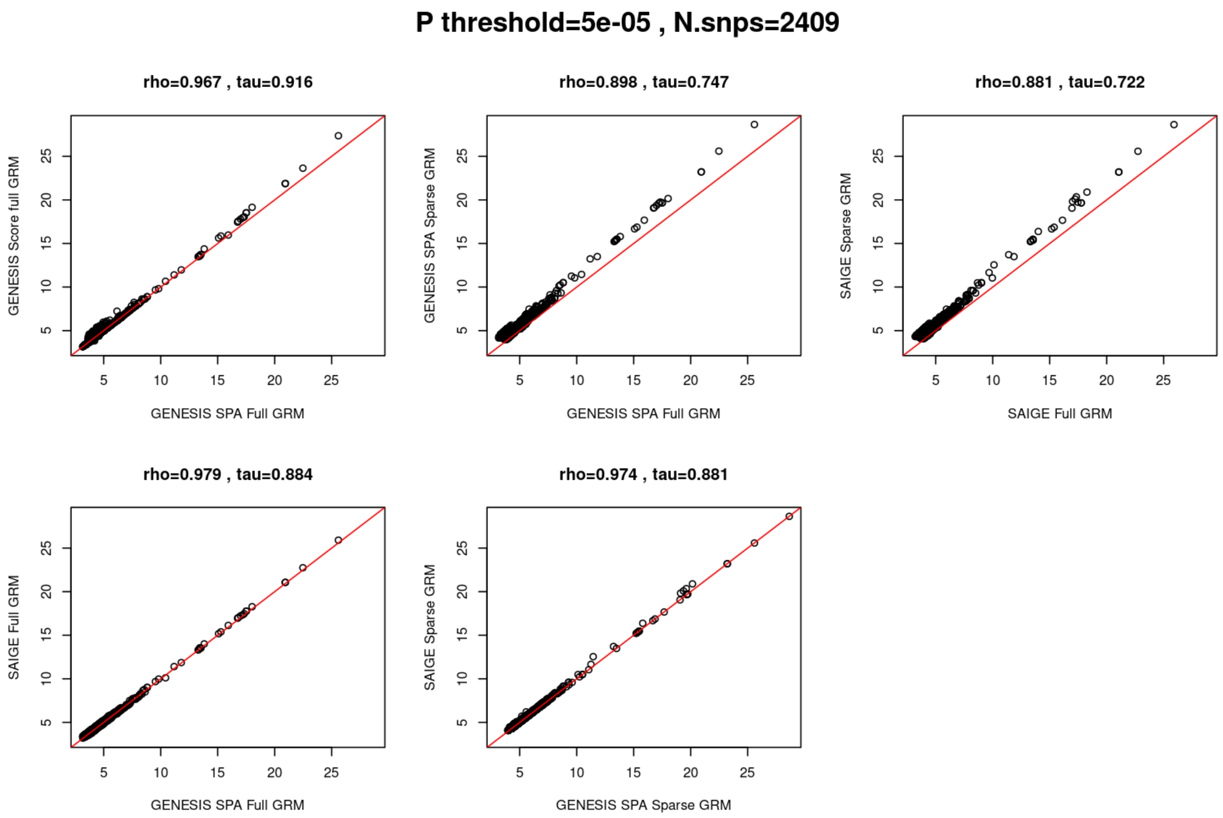
P threshold=5e-05 , N.snps=2409
rho=0.967 , tau=0.916
GENESIS Score full GRM
GENESIS SPA Full GRM
rho=0.898 , tau=0.747
GENESIS SPA Sparse GRM
GENESIS SPA Full GRM
rho=0.881 , tau=0.722
SAIGE Sparse GRM
SAIGE Full GRM
rho=0.979 , tau=0.884
SAIGE Full GRM
GENESIS SPA Full GRM
rho=0.974 , tau=0.881
SAIGE Sparse GRM
GENESIS SPA Sparse GRM

(D)

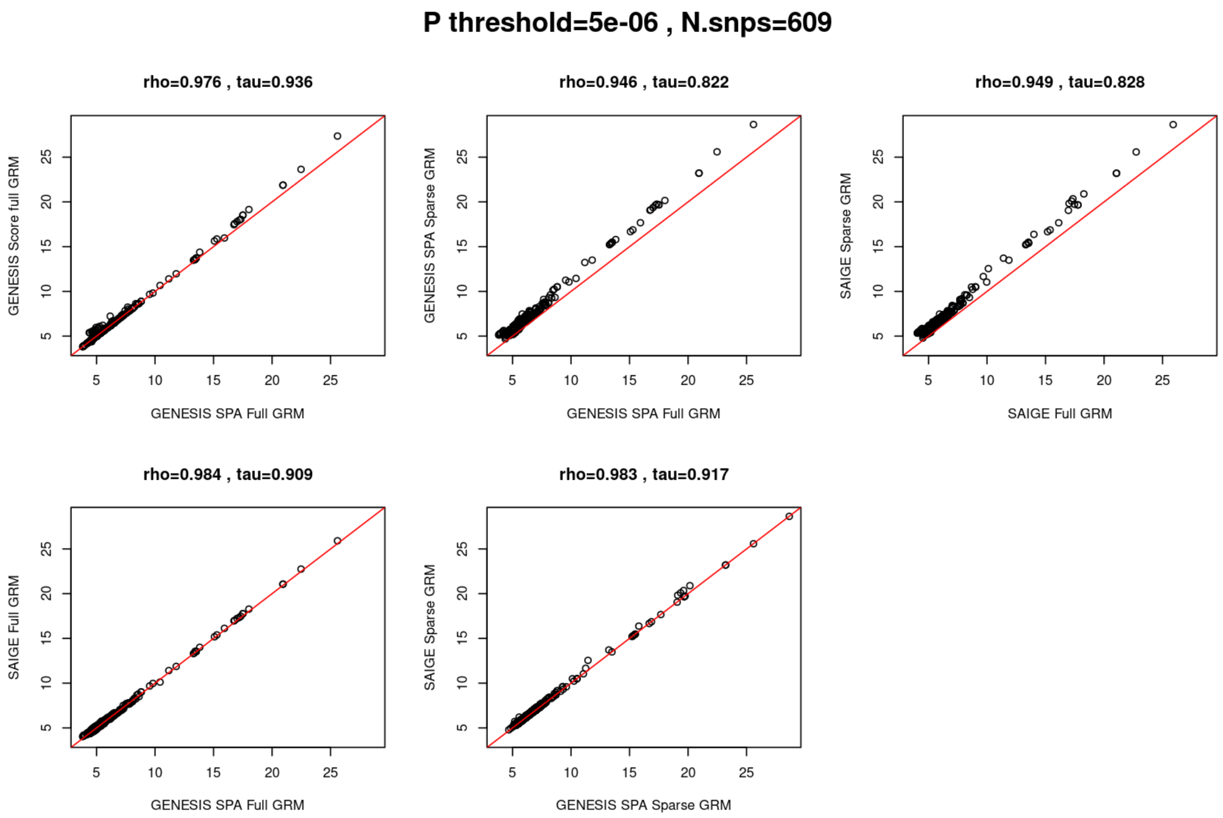
P threshold=5e-06 , N.snps=609
rho=0.976 , tau=0.936
GENESIS Score full GRM
GENESIS SPA Full GRM
rho=0.946 , tau=0.822
GENESIS SPA Sparse GRM
GENESIS SPA Full GRM
rho=0.949 , tau=0.828
SAIGE Sparse GRM
SAIGE Full GRM
rho=0.984 , tau=0.909
SAIGE Full GRM
GENESIS SPA Full GRM
rho=0.983 , tau=0.917
SAIGE Sparse GRM
GENESIS SPA Sparse GRM

(E)

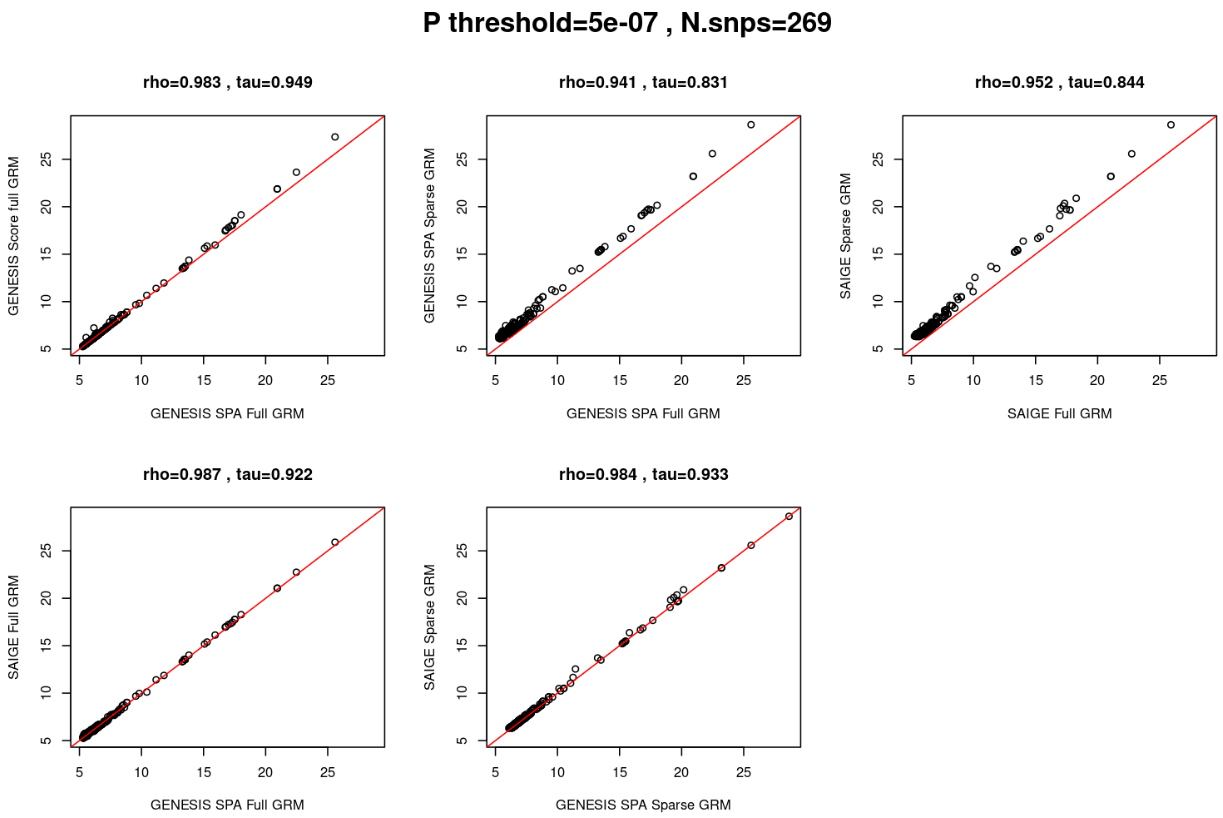
P threshold=5e-07 , N.snps=269
rho=0.983 , tau=0.949
GENESIS Score full GRM
GENESIS SPA Full GRM
rho=0.941 , tau=0.831
GENESIS SPA Sparse GRM
GENESIS SPA Full GRM
rho=0.952 , tau=0.844
SAIGE Sparse GRM
SAIGE Full GRM
rho=0.987 , tau=0.922
SAIGE Full GRM
GENESIS SPA Full GRM
rho=0.984 , tau=0.933
SAIGE Sparse GRM
GENESIS SPA Sparse GRM

(F)

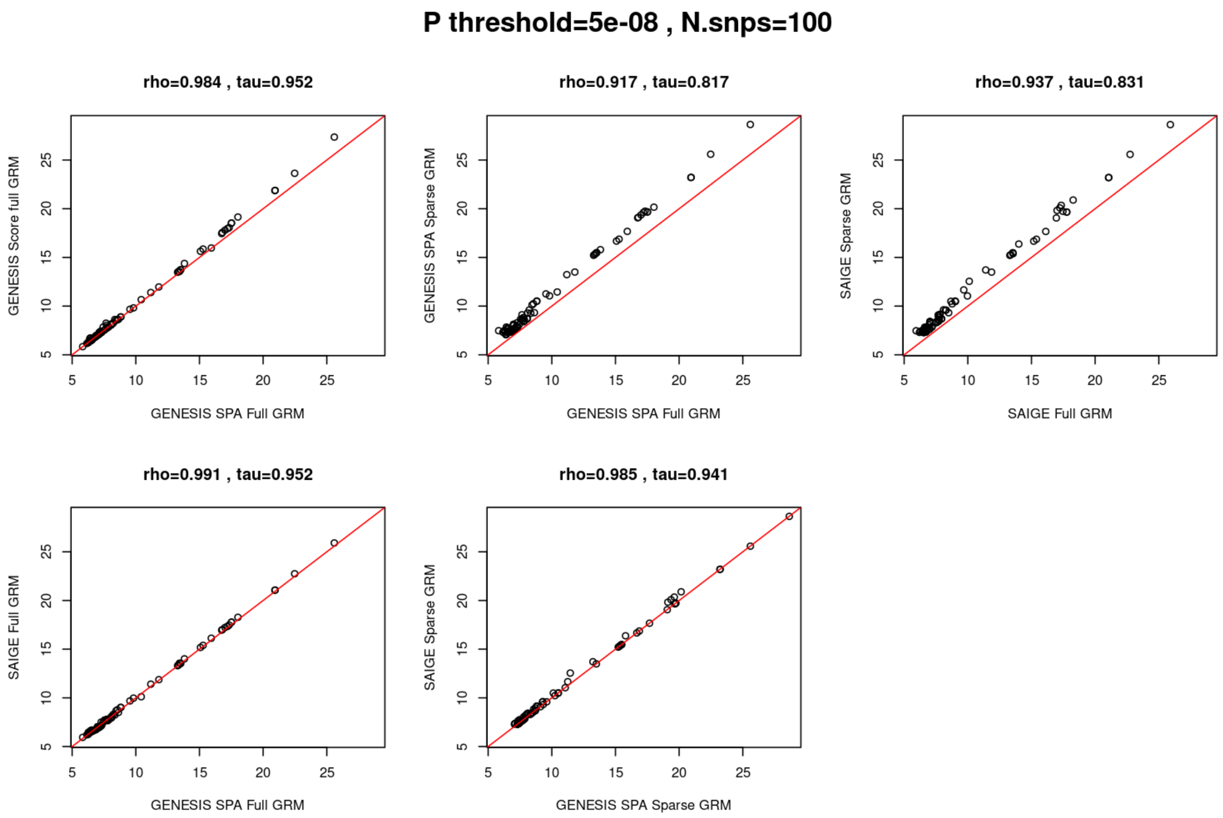

Supplementary Figure 3. Correlations of the ranks of the p-values for WGS data

(A)

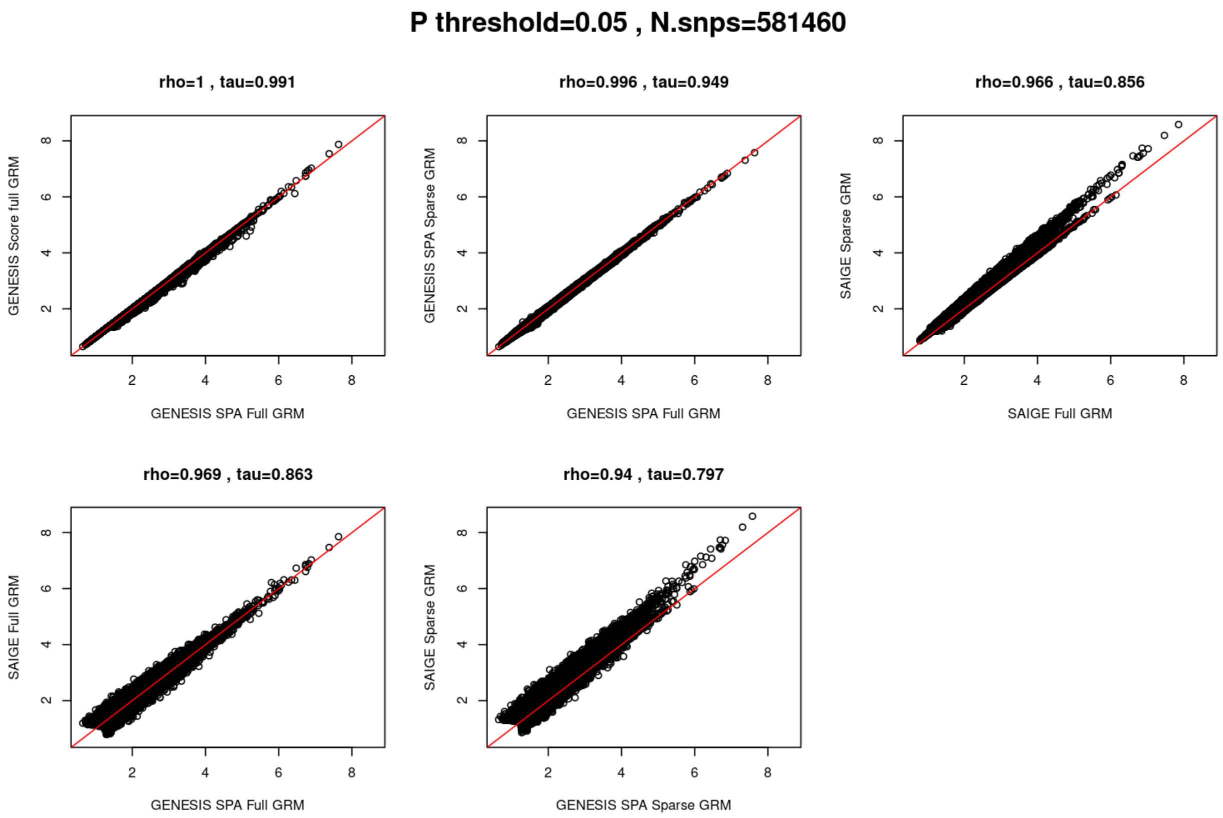
P threshold=0.05 , N.snps=581460
rho=1 , tau=0.991
GENESIS Score full GRM
GENESIS SPA Full GRM
rho=0.996 , tau=0.949
GENESIS SPA Sparse GRM
GENESIS SPA Full GRM
rho=0.966 , tau=0.856
SAIGE Sparse GRM
SAIGE Full GRM
rho=0.969 , tau=0.863
SAIGE Full GRM
GENESIS SPA Full GRM
rho=0.94 , tau=0.797
SAIGE Sparse GRM
GENESIS SPA Sparse GRM

(B)

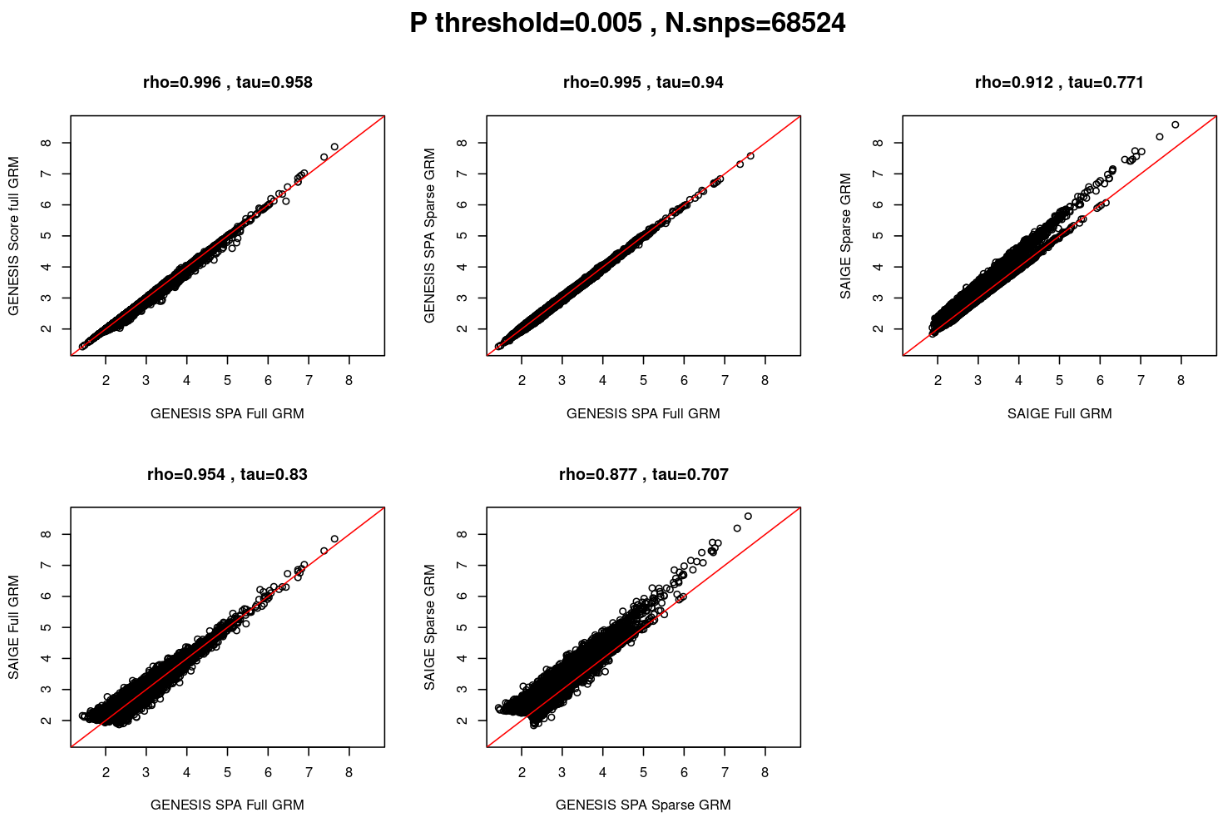
P threshold=0.005 , N.snps=68524
rho=0.996 , tau=0.958
GENESIS Score full GRM
GENESIS SPA Full GRM
rho=0.995 , tau=0.94
GENESIS SPA Sparse GRM
GENESIS SPA Full GRM
rho=0.912 , tau=0.771
SAIGE Sparse GRM
SAIGE Full GRM
rho=0.954 , tau=0.83
SAIGE Full GRM
GENESIS SPA Full GRM
rho=0.877 , tau=0.707
SAIGE Sparse GRM
GENESIS SPA Sparse GRM

(C)

P threshold=5e-04 , N.snps=7757

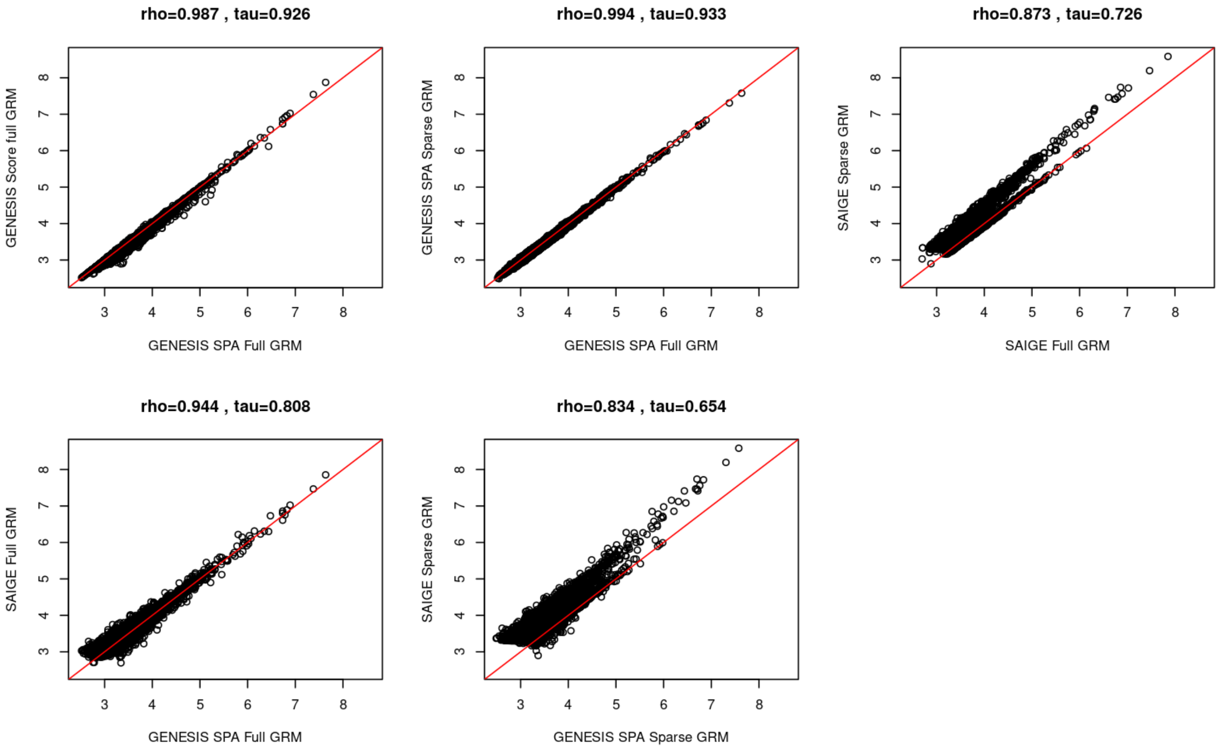

(D)

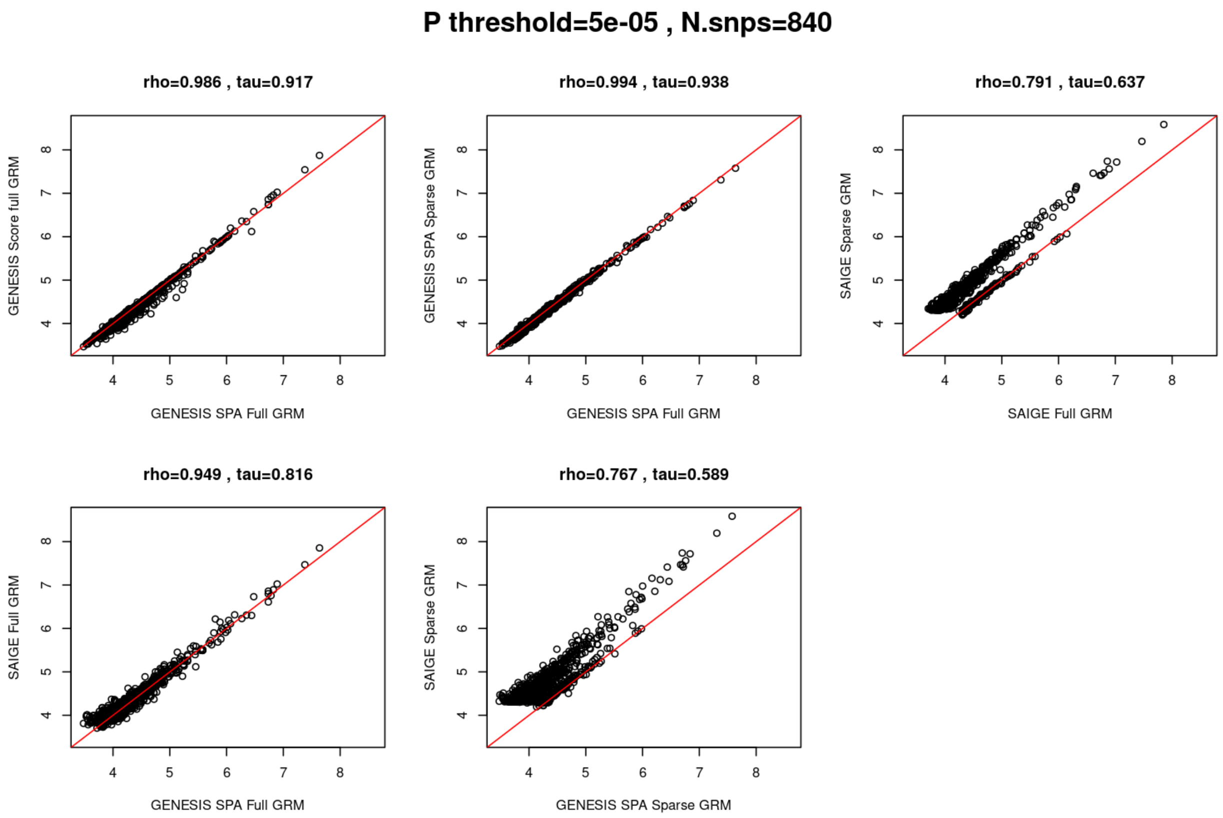
P threshold=5e-05 , N.snps=840
rho=0.986 , tau=0.917
GENESIS Score full GRM
GENESIS SPA Full GRM
rho=0.994 , tau=0.938
GENESIS SPA Sparse GRM
GENESIS SPA Full GRM
rho=0.791 , tau=0.637
SAIGE Sparse GRM
SAIGE Full GRM
rho=0.949 , tau=0.816
SAIGE Full GRM
GENESIS SPA Full GRM
rho=0.767 , tau=0.589
SAIGE Sparse GRM
GENESIS SPA Sparse GRM

(E)

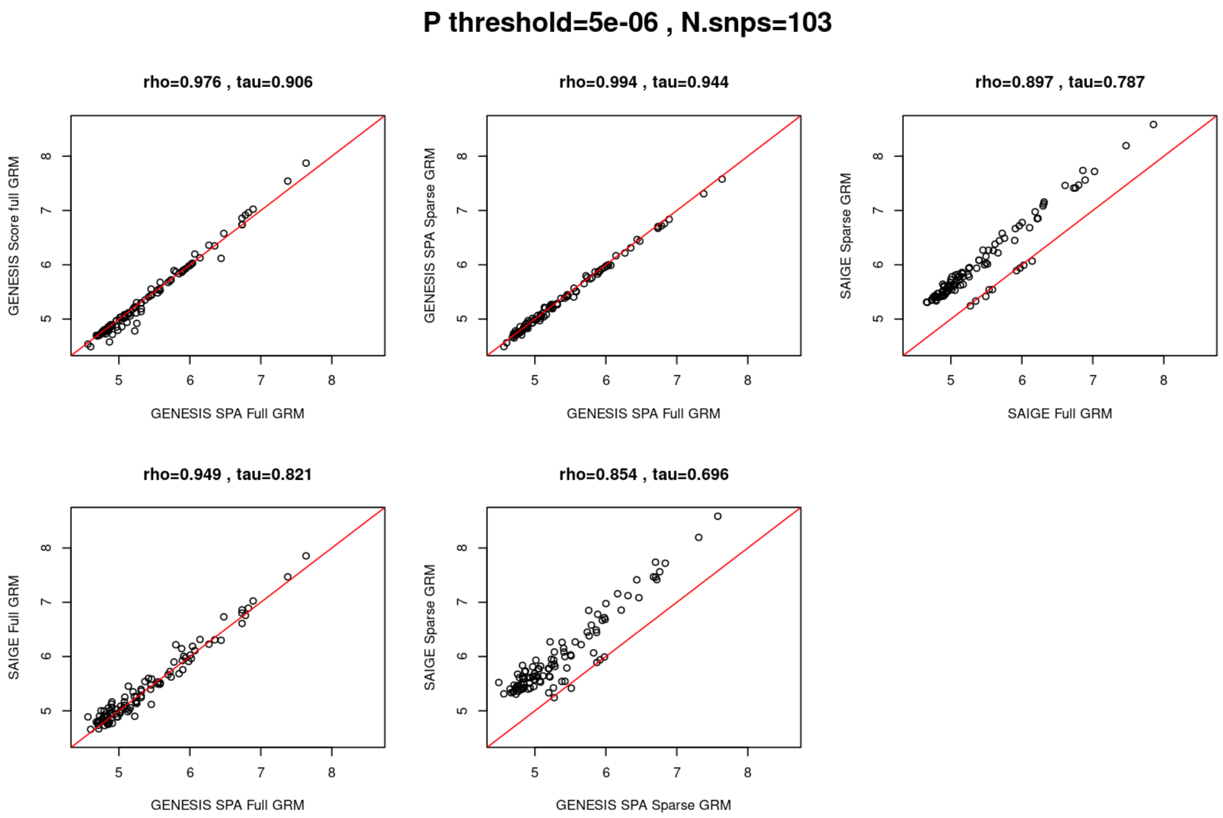

Supplementary Figure 4. <u>Pairwise comparison plots of the Score function values for imputed genotype data</u>

Score function values are denoted in the output from respective software as Tstat and Score in SAIGE and GENESIS.

(A) GENESIS SPA full GRM vs GENESIS Score full GRM (B) GENESIS SPA full GRM vs GENESIS SPA sparse GRM (C) SAIGE full GRM vs SAIGE sparse GRM

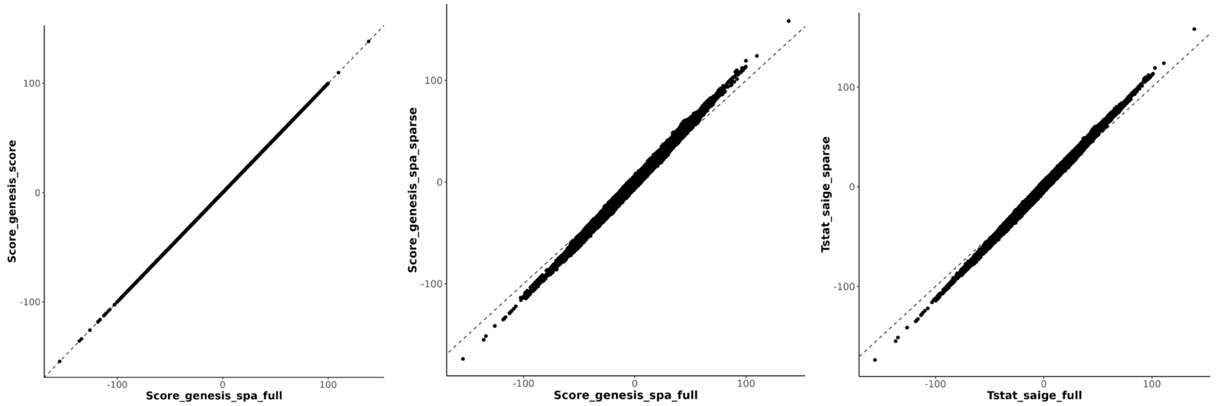

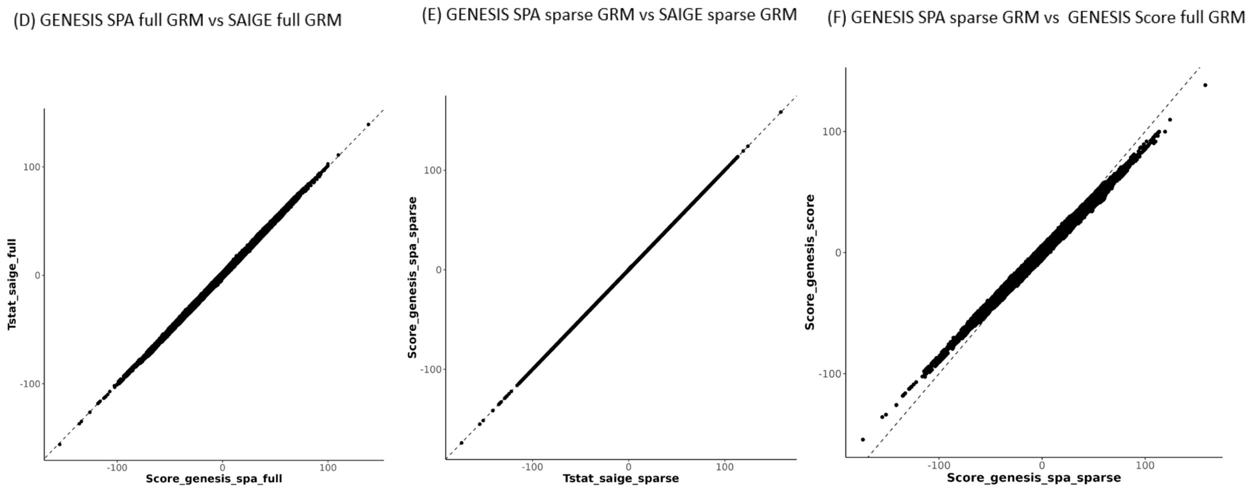

Supplementary Figure 5. <u>Pairwise comparison plots of the Score function values for WGS data</u>

Score function values are denoted in the output from respective software as Tstat and Score in SAIGE and GENESIS.

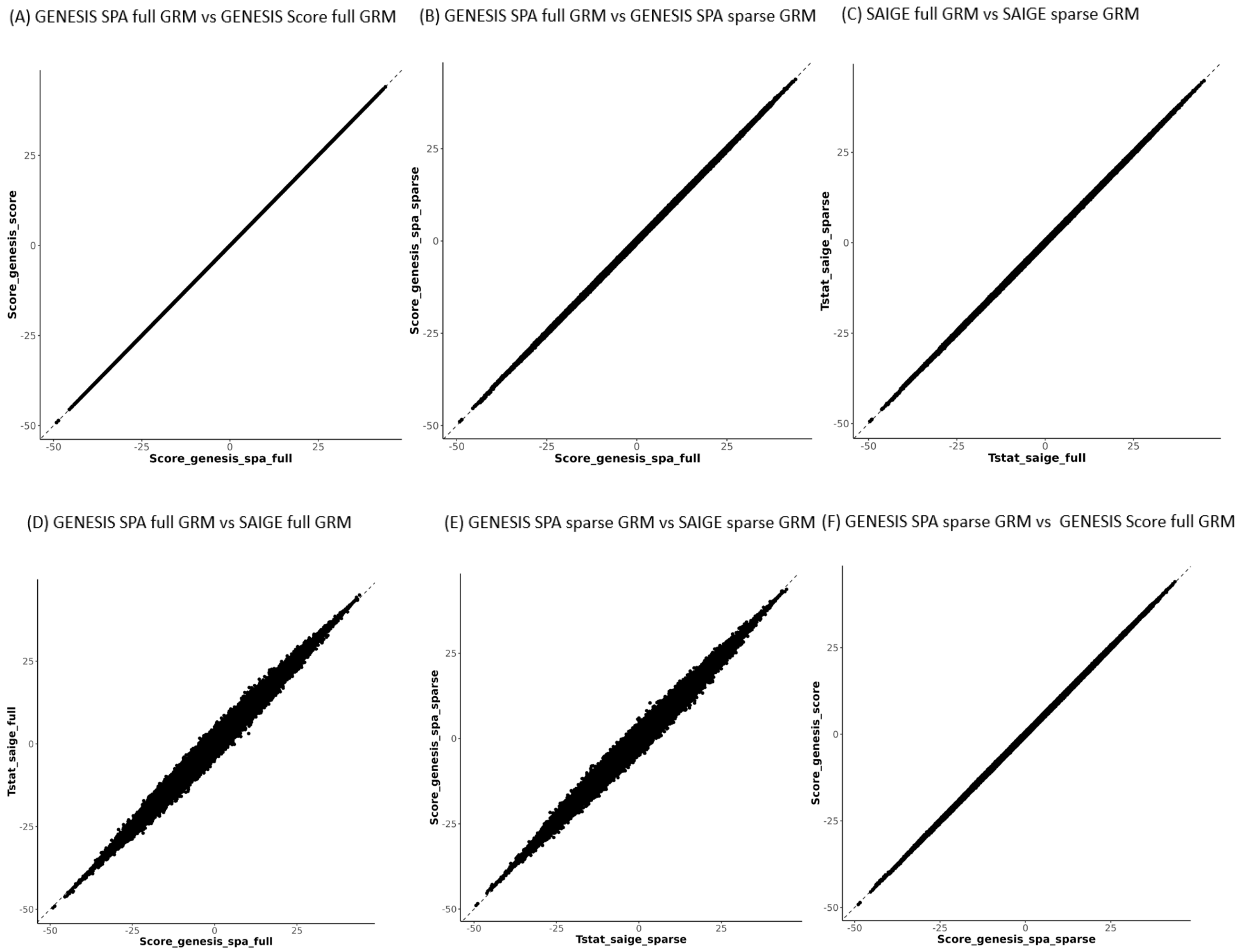

Supplementary Figure 6. **<u>Pairwise comparison plots of the effect estimates for imputed genotype data</u>**

Effect estimates are denoted as BETA in SAIGE, REGENIE and fastGWA-GLMM, and Est in GENESIS.

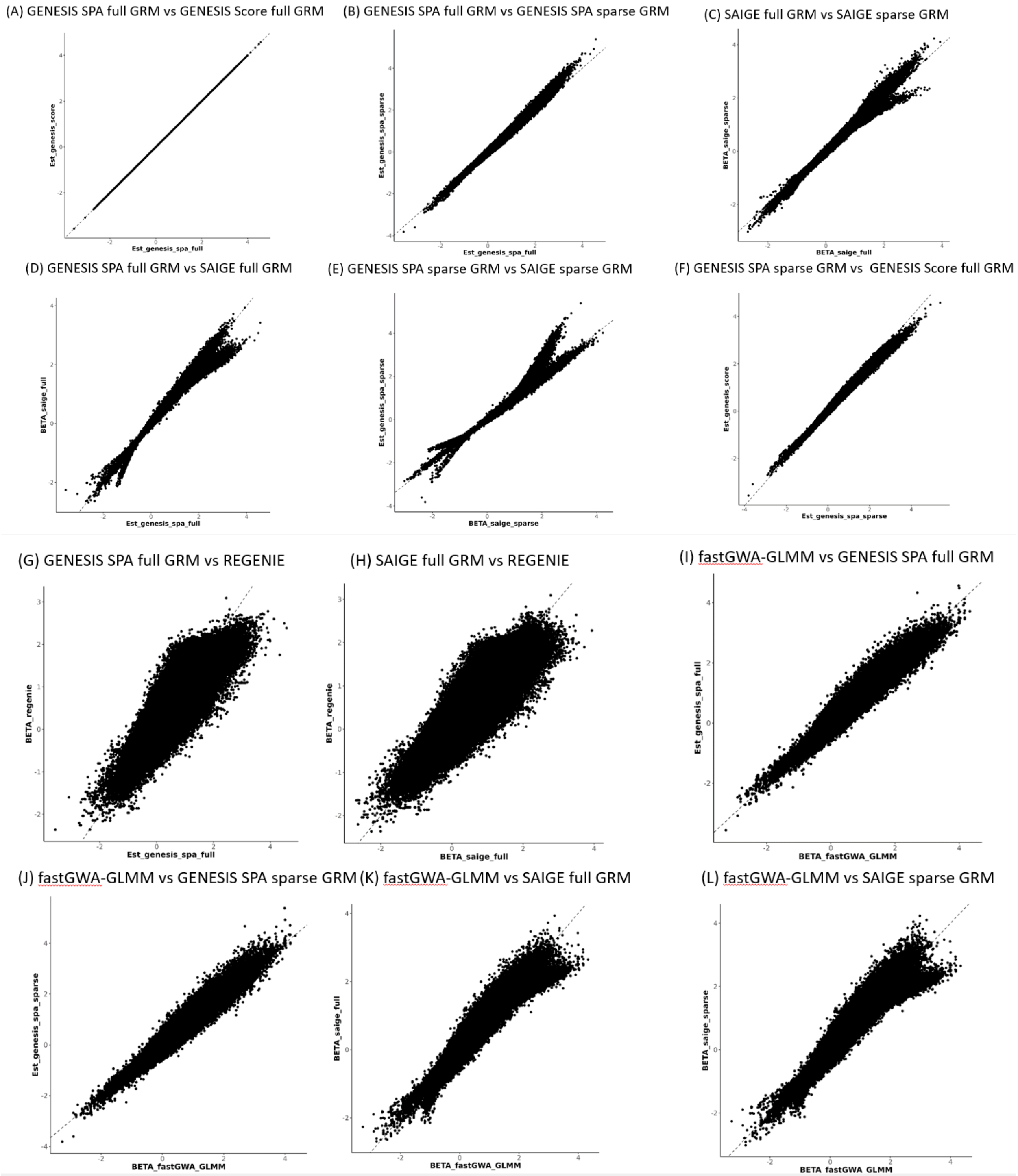

(M) fastGWA-GLMM vs REGENIE

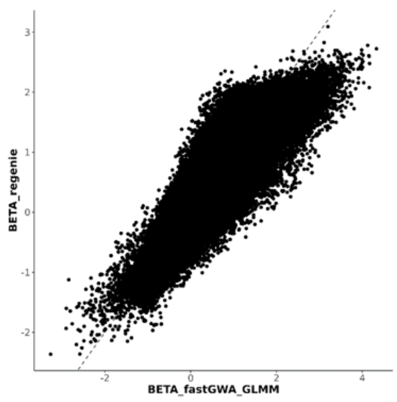

Supplementary Figure 7. **<u>Pairwise comparison plots of the effect estimates for WGS data</u>**

Effect estimates are denoted as BETA in SAIGE, REGENIE and fastGWA-GLMM, and Est in GENESIS.

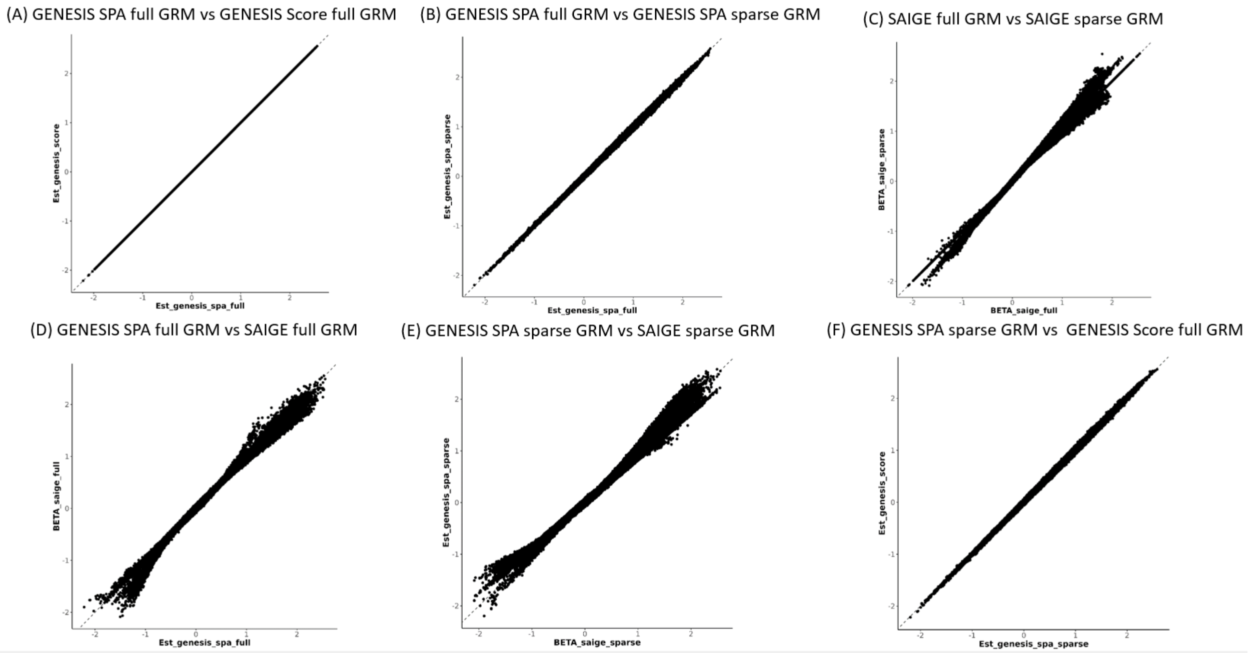

(G) GENESIS SPA full GRM vs REGENIE

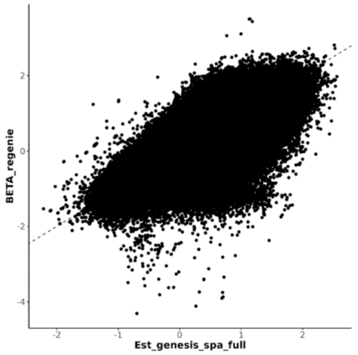

(H) SAIGE full GRM vs REGENIE

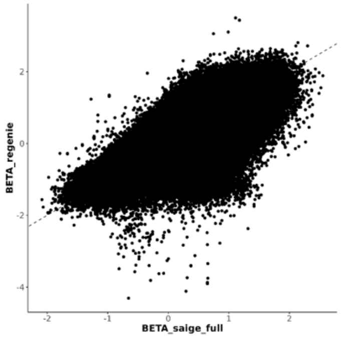

(I) fastGWA-GLMM vs GENESIS SPA full GRM

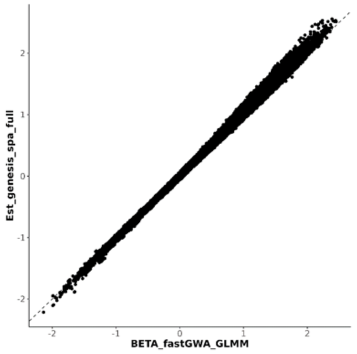

(J) fastGWA-GLMM vs GENESIS SPA sparse GRM

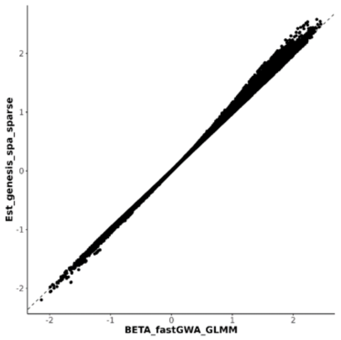

(K) fastGWA-GLMM vs SAIGE full GRM

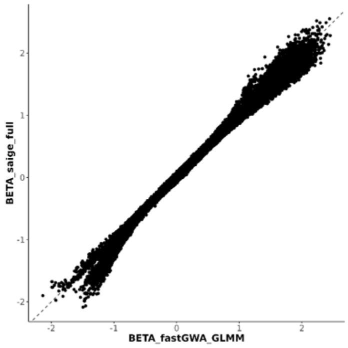

(L) fastGWA-GLMM vs SAIGE sparse GRM

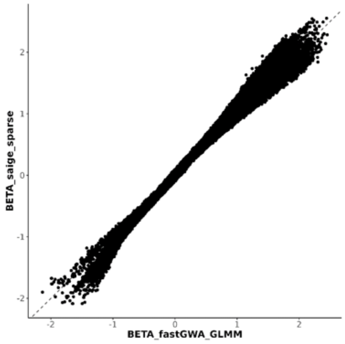

(M) fastGWA-GLMM vs REGENIE

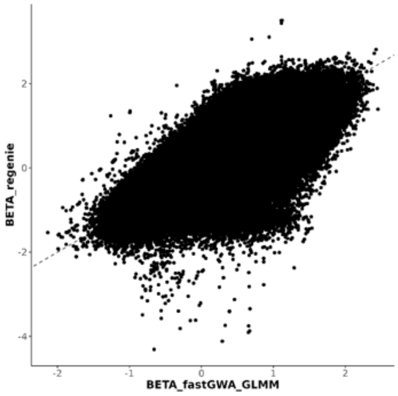

Supplement: Supplementary file 1 [file Presentation1.pdf]
